# Metabolism and biomarkers of heterocyclic aromatic amines in humans

**DOI:** 10.1186/s41021-021-00200-7

**Published:** 2021-07-16

**Authors:** Medjda Bellamri, Scott J. Walmsley, Robert J. Turesky

**Affiliations:** 1 Masonic Cancer Center and Department of Medicinal Chemistry, Cancer and Cardiovascular Research Building, University of Minnesota, 2231 6th Street, Minneapolis, MN 55455 USA; 2 Department of Medicinal Chemistry, College of Pharmacy, University of Minnesota, Minneapolis, MN 55455 USA; 3 Institute of Health Informatics, University of Minnesota, Minneapolis, MN 55455 USA

**Keywords:** Heterocyclic aromatic amines, Mutagens, Cancer, Cooked meat, Metabolism, Biomarkers

## Abstract

Heterocyclic aromatic amines (HAAs) form during the high-temperature cooking of meats, poultry, and fish. Some HAAs also arise during the combustion of tobacco. HAAs are multisite carcinogens in rodents, inducing cancer of the liver, gastrointestinal tract, pancreas, mammary, and prostate glands. HAAs undergo metabolic activation by N-hydroxylation of the exocyclic amine groups to produce the proposed reactive intermediate, the heteroaryl nitrenium ion, which is the critical metabolite implicated in DNA damage and genotoxicity. Humans efficiently convert HAAs to these reactive intermediates, resulting in HAA protein and DNA adduct formation. Some epidemiologic studies have reported an association between frequent consumption of well-done cooked meats and elevated cancer risk of the colorectum, pancreas, and prostate. However, other studies have reported no associations between cooked meat and these cancer sites. A significant limitation in epidemiology studies assessing the role of HAAs and cooked meat in cancer risk is their reliance on food frequency questionnaires (FFQ) to gauge HAA exposure. FFQs are problematic because of limitations in self-reported dietary history accuracy, and estimating HAA intake formed in cooked meats at the parts-per-billion level is challenging. There is a critical need to establish long-lived biomarkers of HAAs for implementation in molecular epidemiology studies designed to assess the role of HAAs in health risk. This review article highlights the mechanisms of HAA formation, mutagenesis and carcinogenesis, the metabolism of several prominent HAAs, and the impact of critical xenobiotic-metabolizing enzymes on biological effects. The analytical approaches that have successfully biomonitored HAAs and their biomarkers for molecular epidemiology studies are presented.

## Introduction

Professor Takashi Sugimura serendipitously discovered mutagens/carcinogens produced in cooked meat and fish over 40 years ago. His wife was broiling fish in the kitchen, and the smoke caught Professor Sugimura’s attention. He reasoned that if cigarette smoke contains many mutagens, why not smoke produced by broiling fish [1, 2]? His observation stimulated research worldwide on cooked food mutagens which led to the identification of more than 20 genotoxic HAAs formed in cooked meat, poultry, and fish [3–8]. Subsequent studies showed that rodents fed HAAs as part of the diet developed cancers in many organs, including the liver, colon, pancreas, breast, and prostate [9–13]. These are common cancer sites in Western countries and are increasing in Japan and other Asian countries adapting Western dietary habits [2]. The subject of dietary mutagens has been an active area of research since Professor Sugimura discovered HAAs and highlighted in his salient review articles over the past five decades [1, 2, 11, 14–17].

### HAA discovery

**Fig. 1:**
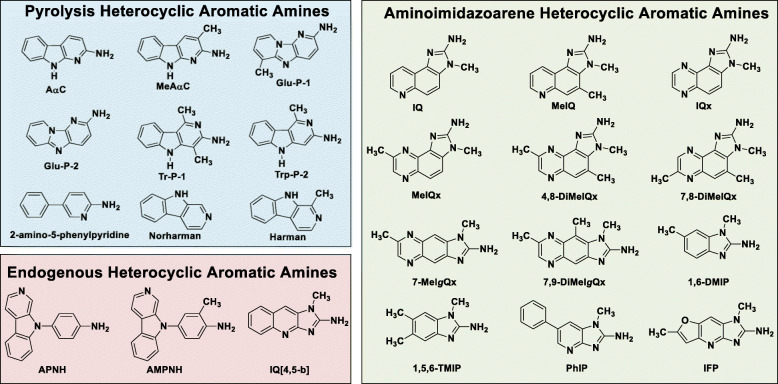
Chemical structures of prevalent HAAs

Professor Sugimura and his colleague Dr. Minako Nago showed that smoke generated by broiled fish was strongly mutagenic in the Ames Salmonella test [18]. Many HAAs are potent bacterial mutagens in the frame-shift tester strain *Salmonella typhimurium* TA98 [1]. The Ames test proved to be an invaluable guide to isolate these novel mutagens from complex cooked food matrices [19]. During the following four decades, many HAAs were identified in cooked foods. The structures of prevalent HAAs are shown in Fig. 1.

### HAA formation

Several HAAs arise during high-temperature pyrolysis of tryptophan, glutamic acid, or proteins to form 3-amino-1,4-dimethyl-5*H*-pyrido[4,3-*b*]indole (Trp-P-1), 3-amino-1-methyl-5*H*-pyrido[4,3-*b*]indole (Trp-P-2), 2-amino-6-methyldiprido[1,2-*a*:3′,2′-*d*]imidazole (Glu-P-1), 2-aminodiprido[1,2-*a*:3′,2′-*d*]imidazole (Glu-P-2), 2-amino-9*H*-pyrido[2,3-*b*]indole (AαC), and 2-amino-3-methyl-9*H*-pyrido[2,3-*b*]indole (MeAαC) [1, 20, 21]. Several pyrolysis HAAs were identified in charred meats and combusted tobacco [3, 22–26]. After these early studies, Kasai and co-workers showed that the highly mutagenic chemicals formed in broiled fish and beef were distinct from the amino acid pyrolysates, which led to the discovery of the second class of HAAs, the aminoimidazoarenes (AIAs), including 2-amino-3-methylimidazo[4,5-*f*]quinoline (IQ), 2-amino-3,4-dimethylimidazo[4,5-*f*]quinoline (MeIQ), and 2-amino-3,8-dimethylimidazo[4,5-*f*]quinoxaline (MeIQx) [27–30]. The Felton laboratory subsequently identified 2-amino-1-methyl-6-phenylimidazo[4,5-*b*]pyridine (PhIP) in fried ground beef [31]. The amounts of HAAs increase in meats as a function of time and cooking temperature; PhIP and AαC levels, in particular, can form at levels up to 50 parts-per-billion (ppb) or higher in well-done cooked meat and poultry compared to other HAAs [3, 4, 25, 32–36]. Pan-fried or grilled scrapings often used for gravies also contain elevated concentrations of HAAs [37, 38]. Other structurally related genotoxic HAAs were subsequently identified in cooked meats, fish, and poultry, including linear tricyclic isomers of the angular tricyclic ring 2-amino-3-methylimidazo[4,5-*f*]quinoxaline (IQx) and its methylated homologs [25, 39, 40].

Since HAAs typically form at the low ppb concentrations in cooked meats, early studies employed model systems to elucidate critical precursors involved in HAA formation. Jägerstad conducted studies with model systems containing amino acids and sugars and showed that creatine or creatinine was the precursor of the imidazo moieties for AIA-type compounds [41]. Later, Murkovic identified phenylacetaldehyde as a critical precursor in PhIP formation [42]. Reactive carbonyl compounds, including acrolein, glucose, and Strecker degradation products are involved in IQ and IQx, and MeIQx formation [41–43].

Recently, Totsuka, Wakabayashi, and co-workers identified several novel HAAs, which are formed from β-carbolines and aniline or *o*-toluidine in biological systems under physiological conditions to produce 9-(4′-aminophenyl)-9*H*-pyrido[3,4-*b*]indole (aminophenylnorharman, APNH) and 9-(4′-amino-3′-methylphenyl)-9*H*-pyrido [3,4-*b*]indole (aminomethylphenylnorharman, AMPNH) [44]. APNH and AMPNH form by the reaction of norharman with the N-hydroxylated metabolites of aniline or *o*-toluidine, resulting in potent genotoxicants [44]. APNH induces hepatic and colon tumors in Fisher-F344 (F344) rats, with high incidence. APNH is detected in the urine of smokers and nonsmokers, indicating its formation occurs endogenously [45]. A linear tricyclic ring isomer of IQ, 2-amino-1-methylimidazo [4,5-*b*]quinoline (IQ[4,5-*b*]), is also formed endogenously in human urine by condensation of creatinine with 2-aminobenzaldehyde [46]. Its genotoxic potential has not been assessed.

### Mutagenesis and carcinogenesis

#### HAA mutagenicity in bacteria

HAAs are potent bacterial mutagens in the Ames frameshift- *Salmonella typhimurium* strains TA97, TA98, and TA1538 but display weaker activity in point mutation TA100 and TA1535 strains (Table 1) [2, 5, 47]. AIAs, including IQ, MelQ, and MelQx, are more potent mutagens than the amino acid pyrolysate HAAs [2, 5, 47]. AIAs induce a CG-dinucleotide deletion in a run of -CGCGCGCG- situated about ten bases upstream from the original *hisD3052* -C- deletion in the frameshift *Salmonella* strain TA1538 [48]. The strain selectivity and the types of mutations induced by HAAs are consistent with HAA adducts formed with guanine [49]. Some HAAs are more potent mutagens in the Ames assay compared to other carcinogens, including aflatoxin B_1_ (AFB_1_) and benzo[*a*]pyrene (B[*a*]P). However, these striking differences in mutagenic potency are not observed in mammalian cells. The potent mutagenicity of HAAs in the *Salmonella typhimurium*-based assays may be attributed to the high expression of esterifying acetyltransferase enzymes, which produce the reactive *N*-acetoxy-HAA intermediates that form DNA adducts [49]. For example, IQ and MeIQx are much weaker mutagens in *Salmonella typhimurium* TA98/1,8DNP6, an *O*-acetyltransferase deficient strain than in YG1024, an *O*-acetyltransferase competent strain [50, 51].

**Table 1:** HAA mutagenicity in *S. typhimurium* tester strains TA98 and TA100^a,b^

HAA	Revertants/μg	
	TA98	TA100
IQ	433,000	7000
MeIQ	661,000	30,000
IQx	75,400	1500
MeIQx	145,000	14,000
4,8-DiMeIQx	183,000	8000
7,8-DiMeIQx	163,000	9900
PhIP	1800	120
Trp-P-1	39,000	1700
Trp-P-2	104,000	1800
Glu-P-1	49,000	3200
Glu-P-2	1900	1200
AαC	300	20
MeAαC	200	120

^a^with rat liver S9 for metabolic activation

^b^reproduced with permission from [2]

Mutagenicity of HAAs has been studied in *Escherichia coli* using *lacZ*, *lacZα*, and *lacI* genes as endpoints. HAA-induced mutations mainly occurred at GC base pairs [52–54]. For example, the MeIQx mutational spectrum in *lacZα* gene is dominated by frameshift mutations (54%) followed by base substitution mutations (41%), while complex mutations represented 5% of the total [54]. Over 90% of the MeIQx-induced mutations occurred at GC base pairs and clustered in two hotspots with runs of three or five GC base pairs. The frameshift mutations were found at position 2576, while base substitutions were mainly observed at position 2532 [54].

#### HAA mutagenicity in mammalian cells

HAA mutagenicity has been studied in Chinese wild-type and repair-deficient hamster ovary (CHO) cells, employing *hprt* and *aprt* loci, [55] and in Chinese hamster lung (CHL) cells at diphtheria-toxin resistance locus [56]. Trp-P-2 showed the highest mutagenic activity in CHL cells, followed by Trp-P-1, MeIQ, IQ, AαC, MeIQx, Glu-P-1, and Glu-P-2. Except for Trp-P-1, all the tested compounds required exogenous metabolic activation [56]. In CHO cells, IQ, MeIQ, and MeIQx induced weak cytotoxic effects, *hprt* mutations, and sister chromatid exchange induction. These effects did not occur in a dose-dependent fashion and only occurred at concentrations that exceeded 10 μg/mL for IQ and 100-800 μg/ml for MeIQ and MeIQx [55, 57]. IQ, MeIQ, and MeIQx-induced cytotoxicity were not altered in the repair-deficient CHO cell line and are likely due to mechanisms other than DNA damage [55, 57]. PhIP, a much weaker bacterial mutagen than IQ, MeIQ, or MeIQx, induced potent cytotoxic effects, *hprt* mutations, sister chromatid exchange, and chromosomal aberrations. These effects were more pronounced in the repair-deficient than the wild-type cell line [55]. PhIP induces GC>TA transversions at the *hprt* locus in human lymphoblastoid cells [58], GC>TA transversions at the *dhfr* gene and AT>TA, CG>AT, and GC>TA transversions at *aprt* in CHO cells [59, 60], and GC>TA transversions at the *hprt* locus in Chinese hamster V79 cells [61]. Frameshift mutations at guanine were also reported and influenced by the base sequence context. In XEMh1A2-MZ, a Chinese hamster cell line genetically engineered to express human cytochrome P450 1A2 (CYP1A2), PhIP induced − 1 G frameshift mutation; 5′-GGGA-3′ to 5′-GGA-3′ [61]. This mutation occurred in the tumor suppressor gene adenomatous polyposis coli (APC) in 50% of PhIP-induced colon tumors in rats [62]. Collectively, these mutations are consistent with HAA-guanine adducts being the primary site of HAA-DNA adduct formation [49, 63, 64].

HONH-PhIP mutagenesis was investigated by whole-genome sequencing (WGS) in human *TP53* knock-in (Hupki) mouse embryo fibroblasts, and mutagenicity in *TP53* and the *lacZ* reporter gene [65]. All HONH-PhIP induced *TP53* mutations occurred at G:C base pairs with CG>TA transversions accounting for most of the mutations; these *TP53* mutations are found in human breast and colorectal tumors cancer types associated with PhIP exposure. *LacZ* mutant frequency increased 25-fold at 5 μM HONH-PhIP, forming the mutation-prone DNA adduct *N*-(2′-deoxyguanosin-8-yl)-PhIP (dG-C8-PhIP) at 350 adducts per 10^8^ nucleotides. A WGS mutational signature defined by CG>TA transversion was present in HONH-PhIP-treated immortalized clones, which showed similarity to COSMIC SBS4, SBS18, and SBS29 mutational signatures found in human tumors [65].

#### HAA mutations in rodents

HAA-induced mutations of oncogenes and tumor-suppressor genes were examined in rodent tumors following long-term feeding studies [66–68]. These studies generally employed the maximum tolerable HAA doses. HAAs induce mutations in several oncogenes and tumor suppressor genes, including *Apc*, Ha-*ras*, Ki-*ras*, *p53*, and *β-Catenin* (Table 2). The *APC* mutation is responsible for familial adenomatous polyposis and also a major rate-limiting and early event in colorectal cancer development [69]. Over half of the PhIP-induced colon tumors in F344 rats harbored a GC base pair deletion in 5′-GTGGGA-3′ at codon 635 of the *APC* gene. This mutation was detected after 1 week of feeding and increased in a dose- and time-dependent manner [62, 70]. This same deletion at 5′-GGGA-3′ occurred in the *lacI* gene of the colon mucosa of the transgenic Big Blue® mice and Big Blue® rats, the *lacI* gene of mammary glands of female Big Blue rats, and in *lacI* gene of the prostate of Big Blue male rats treated with PhIP [71–74]. This GC base pair deletion at the 5′GGGA-3′ sequence may be a signature mutation of PhIP.

**Table 2:** HAA mutagenicity in rodents^a^

HAA	Animal	Strain	Target organ	Mutations								
				H-ras	Ki-ras	N-ras	p53	APC	β-Cat	MM	LacI	LacZ
PhIP	Rat	F344	Colon	0/9	0/9	0/9	0/9	4/8	4/7	7/8		
		Big Blue	Colon mucosa								227/227	
			Mammary gland								149/149	
	Mouse	CDF1	Lymphoid tissue									
		Big Blue	Colon mucosa								115/115	40/40
MeIOx	Rat	F344	Zymbal gland	2/6								
MeIQ	Rat	F344	Zymbal gland	11/14								
IQ	Rat	F344	Colon	0/11	0/11	0/11	0/11	2/13	5/5			
			Zymbal gland	4/7, 5/9	3/9		4/15					
	Mouse	CDF1	Liver	7/34								
			lung		49/54							
Glu-P-1	Rat	F344	Colon	0/7	1/7	0/6	0/7					

^a^reproduced with permission [2, 68]

IQ-induced mutations in the *APC* gene occurred in rat colon tumors but only in 2 out of 13 tumors analyzed [62]. IQ and PhIP induced mutations in *β-Catenin* gene with high frequency in rat colon. Interestingly, the tumors with *β-Catenin* mutations harbored a wild-type *APC,* while tumors with mutated APC harbored a wild-type *β-Catenin.* These data demonstrate the need for only a single mutation in the *β-Catenin/APC* pathway for PhIP-colon carcinogenesis [75]. IQ- and PhIP-induced colon tumors did not harbor mutations in the *ras* genes family [2]. In contrast, Ha-*ras* mutations occurred in PhIP-induced mammary gland tumors of female F344 and Sprague–Dawley rats [76]. Ha-*ras* mutations were also detected in IQ-induced liver tumors in CDF_1_ mice, MeIQ-induced forestomach tumor in CDF_1_ mice, and IQ-, MeIQ, and MeIQx-induced Zymbal’s gland of F344 rats [67, 77–79]. IQ-induced Ki-*ras* mutations in the lung and Zymbal’s gland tumors of CDF_1_ mice and F344 rats, respectively [67, 80]. *p53* mutations infrequently occurred in PhIP-induced mammary gland tumors in F334 rats, MeIQx-induced liver tumors in F334 rats, IQ-induced Zymbal’s gland tumors in F334 rats, and MeIQ-induced forestomach tumors in CDF_1_ mice [2]. IQ induced *p53* mutations in 4 of 20 nonhuman primates hepatic tumors: 3 out of 4 mutations were identified as GC>TA transversions, while the fourth mutation was a GC>AT transition [81] (Table 2). These data suggest that dG-HAA adducts are responsible for the induced mutations [49].

#### HAA carcinogenicity

HAAs are multi-site carcinogens with targeted organs including liver, lung, hematopoietic system, forestomach, and blood vessels in mice, and colon, small intestine, prostate, mammary gland, hematopoietic system, liver, Zymbal’s gland, skin, clitoral gland, oral cavity, and urinary bladder in rats (Table 3) [68, 82, 83]. Some epidemiological studies report that frequent consumption of well-done cooked meat, the primary source of HAA exposure, is linked to breast, colon, and prostate cancer risk [84–89]. It is noteworthy that PhIP is the only HAA and dietary mutagen reported to induce prostate tumors in rodents [13, 90].

**Table 3:** HAA carcinogenicity in animals^a^

HAA	Animal	Strain	Dose (mg/kg)	Exposure peroid (weeks)	Target organ
PhIP	Rat	F344	400	52	Colon, Mammary gland, Prostate, Lymphoid tissue
		Big Blue	400	60	Colon mucosa, Mammary gland
	Mouse	CDF1	400	82	Lymphoid tissue
		C57BL/6 N	300	70-95	Small intestine, Lymphoid tissue
		Big Blue	300		Colon mucosa
MeIOx	Rat	F344	400	61	Liver, Zymbal gland, Clitoral gland, Skin
	Mouse	CDF1	600	84	Liver, lung, Hematopoietic system
MeIQ	Rat	F344	300	40	Colon, Zymbal gland, Skin, Oral cavity, Mammary gland
	Mouse	CDF1	400	91	Liver, Forestomach
		C57BL/6	300		Liver, Colon
		Big Blue	300	60	Colon mucosa
IQ	Rat	F344	300	55 - 72	Liver, Small intestine, Colon, Zymbal gland, Clitoral gland, Skin
	Mouse	CDF1	300	96	Liver, Forestomach, lung
	Monkey	cynomolgus	10 - 20	172 - 240	Liver
MeAαC	Rat	F344	100	100	Liver
	Mouse	CDF1	800	84	Liver, Blood vessels
AαC	Mouse	CDF1	800	98	Liver, Blood vessels
Glu-P-1	Rat	F344	500	64	Liver, Small intestine, Colon, Zymbal gland, Clitoral gland
	Mouse	CDF1	500	57	Liver, Blood vessels
Glu-P-2	Rat	F344	500	104	Liver, Small intestine, Colon, Zymbal gland, Clitoral gland
	Mouse	CDF1	500	84	Liver, Blood vessels
Trp-P-1	Rat	F344	150	52	Liver
	Mouse	CDF1	200	89	Liver
Trp-P-2	Rat	F3444	100	112	Liver, Bladder
	Mouse	CDF1	200	89	Liver

^a^reproduced with permission from [2]

PhIP, MeIQx, and IQ cancer bioassays were also conducted in non-human primates [91]. IQ is a potent hepatocellular carcinogen at doses of 10 or 20 mg/kg in cynomolgus monkeys. The latent period was 60 months with the lowest dose and 43 months with the highest dose. Metastasis of the hepatocellular carcinomas occurred in the lungs of several animals. MeIQx was administered to cynomolgus monkeys at the same doses as for IQ for 84 months but did not induce tumors, only sporadic and non-treatment-related aberrant crypt foci in the colon, glutathione S-transferase pi-positive foci in the liver, and hyperplasia of the lymphatic tissue in the lung and gastrointestinal tract were observed. PhIP also did not induce cancer in cynomolgus monkeys employing the same doses and regimens as for IQ and MeIQx. The wide discrepancy in carcinogenicity between these structurally related HAAs may be attributed to differences in their metabolic activation. Cynomolgus monkeys lack hepatic CYP1A2, which bioactivates all three HAAs [92, 93]. However, IQ is also bioactivated by CYP3A4, CYP2C9, and CYP2C10 isoforms, which may contribute to IQ’s carcinogenicity in the cynomolgus monkey [92]. The marmoset monkey, which constitutively expresses CYP1A2, may be a superior model to the cynomolgus monkey for carcinogenicity studies involving HAAs [93].

#### PhIP prostate carcinogenicity and mechanistic studies in rodents

PhIP induces prostate tumors in the F344 rat [13] and CYP1A-humanized (h-CYP1A) C57BL/6 mice but not in wild-type mice [90], signifying a critical role for human CYP1A2 in PhIP metabolism and tumorigenesis. PhIP forms DNA adducts at high levels in the prostate of Wistar and F344 rats [13, 94, 95] and induces high levels of *lacI* gene mutations in the prostate of the Big Blue® transgenic rat [74, 96]. The dorsolateral prostate lobe of the rodent prostate is homologous to the human peripheral prostate zone, the most common site of prostate cancer (PC) development in humans [97]. PhIP induces inflammation, glandular atrophy, high-grade prostatic intraepithelial neoplasia, and oxidative stress in the prostate of hCYP1A mice and the F344 rat [90, 96, 98, 99]; these are pathology features in common with human PC [97]. PhIP treatment increased the expression of the androgen receptor (AR), a key regulator for prostate cell proliferation, increased expression levels of Ki-67, a marker of cell proliferation, and increased expression levels of COX-2, a cyclooxygenase that catalyzes the formation of pro-inflammatory prostaglandin in the prostate of rats and h-CYP1A mice [90, 96, 99]. PhIP also decreased the levels of Nuclear Factor (erythroid-derived 2)-like 2 (Nrf2), a transcription factor of several cytoprotective proteins, E-Cadherin, an epithelial cell adhesion protein, and also decreased levels of p63, phosphatase, and tensin homolog (PTEN), key players key players in cell proliferation and apoptosis [90, 96, 99]. These features are hallmarks for human PC and serve as diagnostic criteria for this malignancy [100–103]. The mutagenic, carcinogenic, and molecular events induced by PhIP in rodent prostate strengthen the unconfirmed paradigm of PhIP playing a role in cooked red-meat related PC etiology [87]. However, the doses of PhIP used in animal studies were a million-fold or higher than the daily intake of PhIP in the human diet. Thus, studies at dietary PhIP exposure levels are warranted to understand the biological effects and potential role of PhIP in human PC.

### Metabolism: bioactivation and detoxication of HAAs

#### Phase I and phase II enzymes

**Fig. 2:**
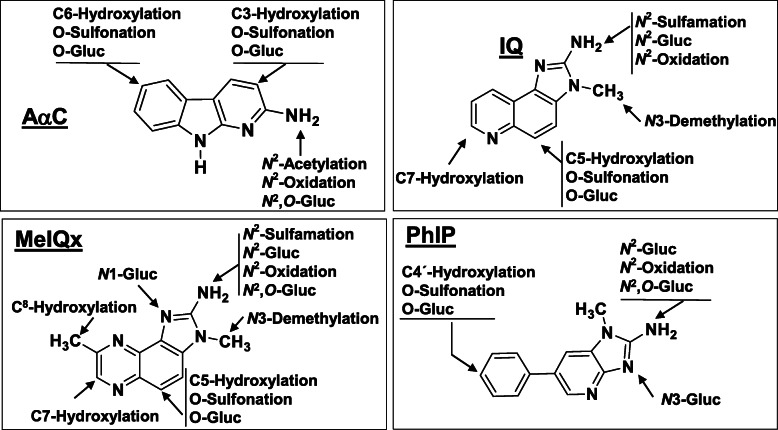
Major pathways AαC, IQ, MeIQx, and PhIP metabolism in experimental laboratory animals and humans

The metabolism of several prevalent HAAs have been characterized in rodents, non-human primates, and humans (Fig. 2). Both phase I and II enzymes contribute to HAA metabolism.

#### Cytochrome P450 oxidation of HAAs

The bioactivation of HAAs occurs by N-oxidation of the exocyclic amine groups to produce the HONH-HAAs. The liver displays the highest metabolic activity in rodents, particularly after enzyme induction with 3-methylcholanthrene or polychlorinated biphenyl [64, 104]. The HONH-HAAs are genotoxic metabolites that covalently bind to DNA to form mutation-prone DNA adducts [63, 64]. Hepatic cytochrome (CYP) CYP1A2 and extrahepatic CYP1A1 and CYP1B1 are principal CYPs that catalyze HAA N-oxidation in humans [64, 93, 105–108]. A pharmacokinetic study estimated that CYP1A2 accounts for 91% of the metabolism of MeIQx and 70% of the PhIP metabolism in humans [105]. The major CYP1A2 pathway of PhIP metabolism occurs through N-oxidation to form the genotoxic *N*-hydroxy-2-amino-1-methyl-6-phenylimidazo[4,5-*b*]pyridine (HONH-PhIP) metabolite, whereas the major CYP1A2 pathway of MeIQx metabolism is through oxidation of the 8-methyl group to form the detoxicated product 2-amino-3-methylimidazo[4,5-*f*]quinoxaline-8-carboxylic acid (IQx-8-COOH) [49, 109].

**Fig. 3:**
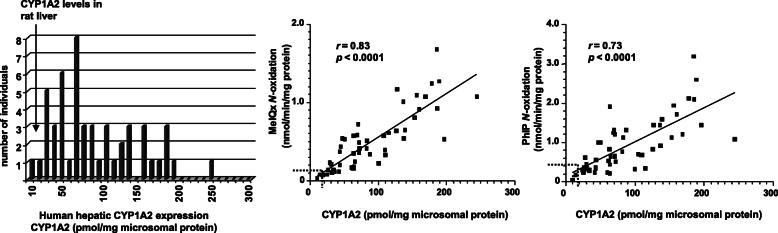
Levels of CYP1A2 expression in rat and human liver microsomes correlates with CYP1A2 expression, MeIQx, and PhIP N-oxidation rates. The checkered lines depicted in the regression curves show the upper levels of CYP1A2 expression and MeIQx and PhIP N-oxidation rates in rat liver microsomes. Adapted with permission from [107]

**Fig. 4:**
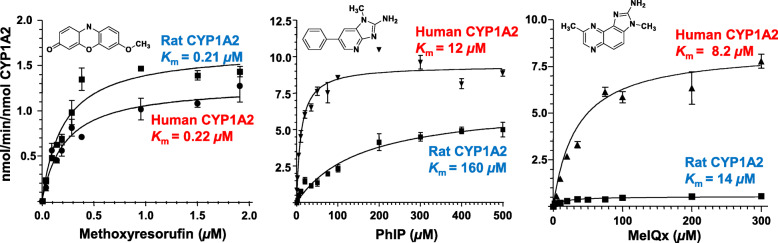
Kinetic parameters of MeIQx, PhIP N-oxidation, and methoxyresoruin oxidative demethylation by rat and recombinant human CYP1A2

There are critical interspecies differences in HAA metabolism by CYPs among rodents, nonhuman primates, and humans that are mainly attributed to different levels of CYP expression, differences in catalytic activities, and regioselectivities of CYPs towards HAAs (Figs. 2 and 3) [93, 107, 110–113]. CYP1A2 expression in the human liver ranges from 5 to 300 pmol per mg of microsomal protein, whereas CYP1A2 expression in non-induced rat liver typically ranges between 10 and 35 pmol/mg microsomal protein in various rat strains and undetected in the cynomolgus monkeys (< 1 pmol/mg microsomal protein) used in carcinogenesis bioassays (Fig. 3) [2, 93, 107, 114–116]. CYP1A2 expression in human liver microsomes is highly correlated to MeIQx and PhIP N-oxidation rates (Fig. 3) [107]. Compared to human liver microsomes, rat and cynomolgus monkey liver microsomes poorly bioactivate IQ, MeIQx, and PhIP to their genotoxic HONH-HAA intermediates because of low CYP1A2 expression but efficiently catalyze HAA ring oxidation, a detoxication pathway, whereas CYPs in human liver microsomes poorly carry out this reaction (Fig. 2) [92, 93, 107, 110–113, 117]. Human CYP1A2 catalyzes IQx-8-COOH formation, the predominant detoxicated metabolite of MeIQx formed in human hepatocytes [109, 118], and IQx-8-COOH is the major urinary metabolite of MeIQx in the urine of meat-eaters [119–122]. Rat CYPs do not catalyze IQx-8-COOH formation (Fig. 2). Recombinant human CYP1A2 shows catalytic efficiencies of MeIQx and PhIP N-oxidation 10-19-fold higher than rat CYP1A2 [107, 117]. In contrast, both human and rat CYP1A2 orthologs display similar catalytic efficiency in the *O*-demethylation of 7-methoxyresorufin (Fig. 4). These interspecies distinctions in enzyme expression and activities impact the biological effects of HAAs and must be considered when assessing the human health risk of HAAs based on experimental animal toxicity data [107, 118, 123].

#### Phase II metabolism of HAAs

Phase II enzymes have a dual role in HAA metabolism, contributing to detoxication and bioactivation. Some HAAs undergo detoxication through direct conjugation of their exocyclic amine groups by phase II enzymes, including N-acetyltransferases (NATs), sulfotransferases (SULTs), and uridine 5′-diphosphoglucuronosyltransferases (UGTs) [49]. NAT1 and NAT2 are two distinct N-acetyltransferase isoenzymes. NAT2 is expressed primarily in the liver, whereas NAT1 is prominently expressed in extrahepatic tissues [124]. Many structurally related carcinogenic aromatic amines undergo by NATs [125, 126]. Glu-P-I, Glu-P-2, and AαC undergo extensive N-acetylation [127–130]; however, AIAs are poor substrates for NATs [49]. Human sulfotransferases (SULTs) belong to a superfamily of genes that are divided into two subfamilies: the phenol SULTs (SULT1) and the hydroxysteroid SULTs (SULT2). SULT1A1, 1A3, and 1B1 are expressed in all parts of the gastrointestinal tract, often exceeding the protein levels expressed in the liver [131–134]. In addition to sulfating phenolic xenobiotics, steroids, and estrogens, SULTs contribute to the detoxication and bioactivation of HAAs. IQ and MeIQx form sulfamic acid derivatives as prominent detoxication metabolites in rats and catalyzed by SULT1A1 [135–137]. The MeIQx sulfamate *N*^2^-(3,8-dimethylimidazo[4,5-*f*]quinoxalin-2-yl-sulfamic acid (MeIQx-*N*^2^-SO_3_H) is detected in human urine [119]. Other HAAs do not undergo sulfamation in rodents or humans; however, sulfate conjugates of HAA ring-hydroxylated metabolites occur, particularly in rodents and nonhuman primates [49, 92, 138]. The UGTs are subdivided in the 1A, 2A, and 2B subfamilies and expressed in liver and extrahepatic tissues, and play a major role in drug and steroid metabolism [139, 140]. UGTs catalyze *N*^2^-glucuronide (Gluc) formation of IQ, PhIP, AαC, *N*1- and *N*^2^-Gluc conjugates of MeIQx, and *N*^2^- and *N*3-Gluc conjugates of PhIP [49, 138, 141, 142]. The UGT1A enzyme family is principally involved in the N-Gluc of PhIP [143] and most likely MeIQx in humans [109]. The Gluc conjugates of PhIP and MeIQx are excreted in meat-eaters’ urine [119, 144].

NATs, SULTs, and kinases catalyze the bioactivation of HONH-HAAs through the formation of highly reactive esters to produce the penultimate species that covalently bind DNA [63, 64, 111, 145]. Most HONH-HAAs undergo bioactivation by human NAT2 [133, 146–149], although both isoforms bioactivate HONH-AαC [128]. The *N*-acetoxy intermediates of *N*-hydroxyamino-3,8-dimethylimidazo[4,5-*f*]quinoxaline (HONH-MeIQx), *N*-hydroxy-2-amino-3-methylimidazo[4,5-*f*]quinoline (HONH-IQ), and *N*-hydroxyamino-9*H*-pyrido[2,3-*b*]indole (HONH-AαC) are short-lived and cannot be isolated [150, 151]. However, *N*-acetoxy-PhIP is sufficiently stable for HPLC isolation and characterization by mass spectrometry, allowing distinction between *N*-acetoxy-PhIP and its hydroxamic acid isomer [152, 153]. Human SULT1A1 and SULT1A2 catalyze the binding of the *N*-hydroxy metabolites of PhIP, AαC, and MeAαC to DNA; the *N*-hydroxy metabolites of MeIQx and IQ are poor substrates for both SULT isoforms [154–157].

**Fig. 5:**
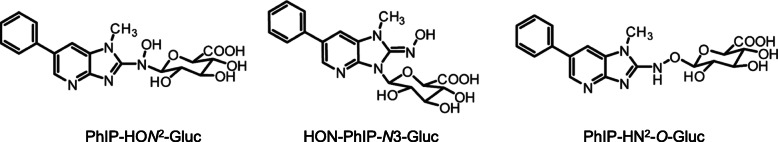
Chemical structures of HONH-PhIP Gluc conjugates

**Fig. 6:**
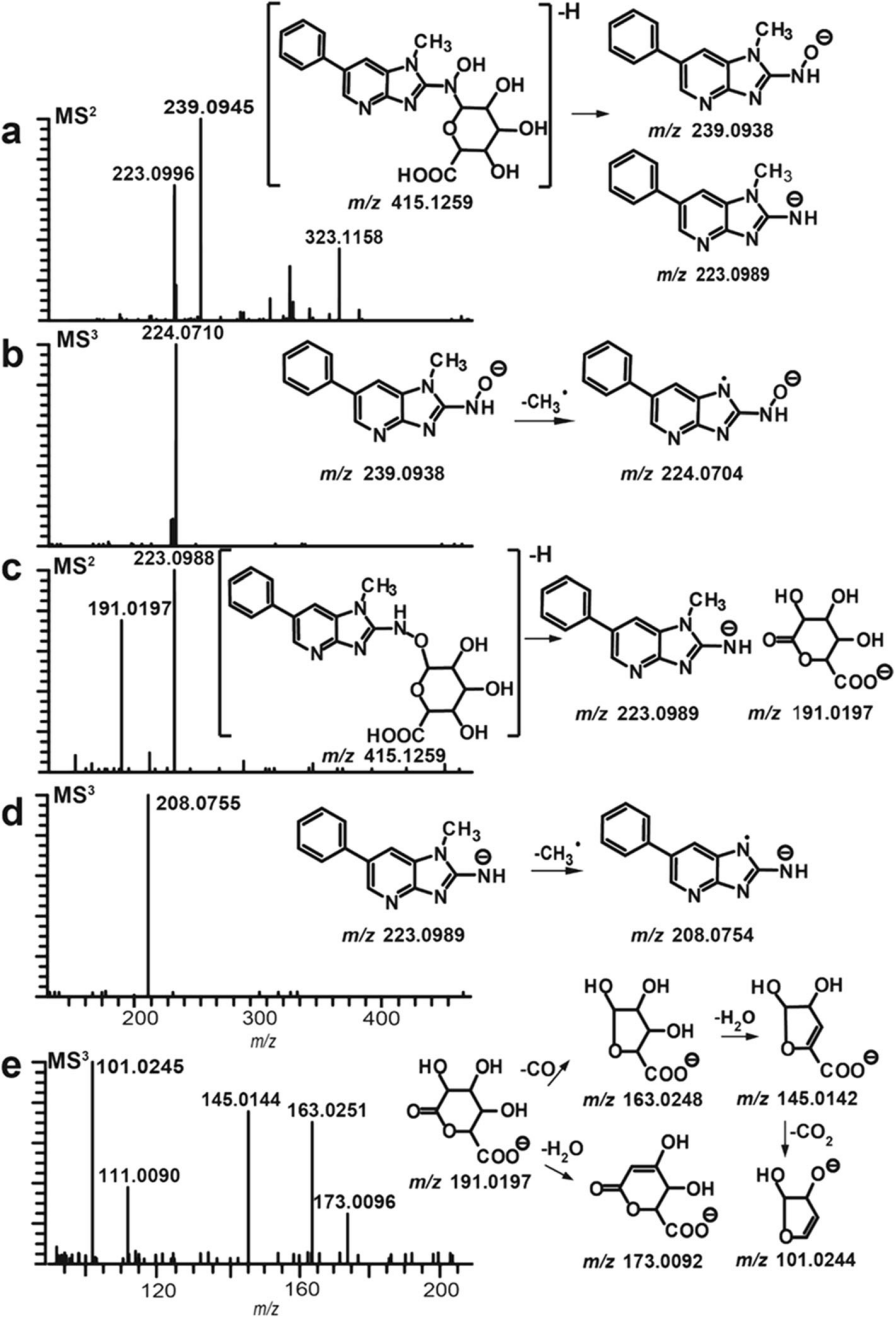
Characterization of isomeric HONH-PhIP Gluc conjugates by multistage scanning (MS^n^) with an Orbital trap by electrospray ionization in the negative ion mode. **a** PhIP-HO*N*^2^-Gluc, MS^2^ at *m/z* 415.1259, **b** MS^3^ at 415.1259 > 239.0938 >, **c** PhIP-HN^2^-*O*-Gluc; MS^2^ at *m/z* 415.1259, **d** MS^3^ at 415.1259 > 223.0989 >, and **e** MS^3^ at *m/z* 415.1259 > 191.0197 >). Adapted with permission from [160]

Gluc conjugation of HONH-HAAs occurs at the exocyclic amine group to form proposed detoxication metabolites [158]. Human liver microsomes catalyze Gluc formation of *N*-hydroxy-HAAs of IQ, MeIQx, PhIP, and AαC [142, 158–160]. Many of these metabolites are excreted in urine of meat-eaters [49, 120, 122, 144, 161]. UGTs 1A1, 1A4, and 1A9 catalyze Gluc of HONH-PhIP [158, 159, 162–164]. *N*^2^-(β-D-glucosiduronyl)-2-hydroxyamino-1-methyl-6-phenylimidazo[4,5-*b*]pyridine (PhIP-HO*N*^2^-Gluc) is the major HONH-PHIP Gluc conjugate formed in rat hepatocytes and with human liver microsomes [158, 162]. A second isomeric Gluc conjugate of HONH-PhIP forms, but the limited quantity of material precluded its characterization by NMR [158]. The site of Gluc conjugation was proposed to occur at the *N*3 imidazole atom of the oxime tautomer of HONH-PhIP (HON-PhIP-*N*3-Gluc) (Fig. 5), based on comparison of UV spectral and biochemical properties to those of the *N*^2^- and *N*3-Gluc conjugates of PhIP [141, 158]. Our laboratory further characterized both HONH-PhIP Gluc metabolites by high-resolution accurate mass spectrometry [160]. The product ion spectra acquired in the negative ion mode clearly showed that the minor Gluc metabolite was conjugated to the oxygen atom of HONH-PhIP to produce *O*-(β-D-glucosiduronyl)-2-hydroxyamino-PhIP (PhIP-HN^2^-*O*-Gluc) (Fig. 6) and not conjugated to the *N*3 imidazole atom of the oxime as was originally proposed (Fig. 5) [158]. Recombinant UGT1A1 and UGT1A4 preferentially catalyze PhIP-HO-*N*^2^-Gluc, while UGT1A9 produced greater amounts of PhIP-HN^2^-*O*-Gluc [164].

HONH-AαC, the genotoxic metabolite of AαC, also forms an *O*-linked Gluc conjugate, *O*-(β-D-glucosiduronyl)-2-hydroxyamino-9*H*-pyrido[2,3-*b*]indole (AαC-HN^2^-*O*-Gluc), as the primary conjugate produced by human liver microsomes and lesser amounts of *N*^2^-(β-D-glucosiduronyl)-2-hydroxyamino-9*H*-pyrido[2,3-*b*]indole (AαC-HO*N*^2^-Gluc) are formed [142, 160]. Recombinant UGT1A9 is the most catalytically efficient UGT isoform in producing AαC-H*N*^2^-*O*-Gl and has the lowest apparent *Km* (0.7 μM) of the UGTs assayed. Studies with human hepatocytes confirmed the preferred formation of AαC-HN^2^-*O*-Gluc over AαC-HO*N*^2^-Gluc [142].

The AαC-HN^*2*^-*O*-Gl conjugate is a biologically reactive metabolite that binds to DNA in vitro under physiological pH at ~ 50-fold higher levels than HONH-AαC; the PhIP-HN^2^-*O*-Gluc binding to DNA was about 3-fold greater than for HONH-PhIP [142]. In contrast, the PhIP-HO*N*^2^-Gluc and AαC-HO*N*^2^-Gluc are weakly bound to DNA [160]. Thus, UGT-mediated *O*-glucuronidation is a possible bioactivation mechanism for HONH-AαC and HONH-PhIP. UGT-mediated Gluc conjugation of chemicals is considered a detoxication mechanism; however, UGTs contribute to the bioactivation of some NSAIDs to form acyl Gluc conjugates [165] and N-*O*-glucuronides of arylhydroxamic acids (*N*-hydroxy-*N*-acetylarylamines), where the electrophilic nitrenium ion species can react with protein or DNA [166–168].

In one population-based case-control study investigating individuals who frequently ate cooked meat, subjects with genetic variations in UGT1A1 harboring functional variants with intermediate or low enzyme activity, based on genotype, were at greater risk for colon cancer than high activity genotype individuals [169]. Individuals with low UGT1A1 functional activity may be at elevated risk because they less efficiently detoxicate HAA or PAH procarcinogens ingested from cooked meats than subjects with rapid UGT1A1 activity. However, the authors reported a paradox with UGT1A9. The strongest association for colon cancer risk in meat-eaters was observed among the high/intermediate UGT1A9 genotype, indicating that either poor genotype-phenotype correlation or some other chemical(s) formed in pan-fried red meat other than HAAs was driving the association. Our biochemical data on UGT1A9 activity offers an alternative interpretation. The high/intermediate UGT1A9 genotype individuals can bioactivate HONH-HAAs, such as HONH-AαC and HONH-PhIP, to produce HAA-HN^2^-*O*-Gluc metabolites in the liver, followed by their elimination via the bile to the gastrointestinal tract, where ensuing binding to colonic DNA can occur [160]. Given the prominent role of UGTs in HAA metabolism, further studies are warranted to assess UGT genetic polymorphisms in the cancer susceptibility of meat-eaters.

**Fig. 7:**
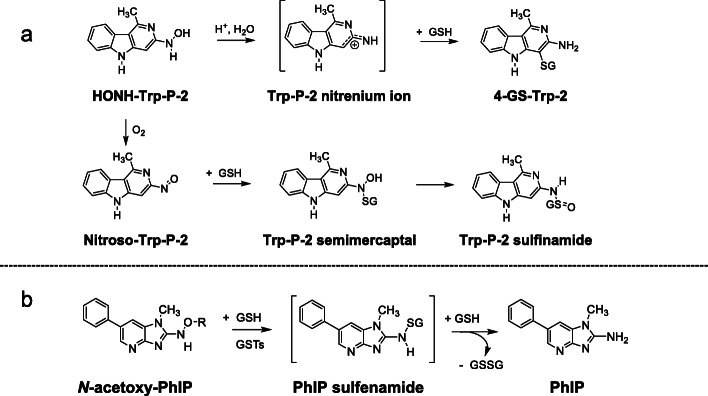
Reaction pathways of **a** HONH-Trp-P-2 and **b** *N*-acetoxy-PhIP intermediates with GSH and GSTs

The Glutathione S-transferases (GST) are another important class of enzymes involved in the detoxication of many endogenous electrophiles and xenobiotics, including HAAs [170]. Human GST enzymes are classified as Alpha (GSTA), Mu (GSTM), Omega (GSTO), Pi (GSTP), Theta (GSTT) and Zeta (GSTZ) [171]. Glutathione (GSH) reacts non-enzymatically or catalyzed by GST to detoxify HONH-HAAs and their reactive N-O-esters. GSH reacts with the nitroso metabolite of Glu-P-1 in vitro to form glutathione sulfinamide and sulfonamide adducts [172]. GSTs in rat liver catalyzed the formation of three GSH conjugates with the *N*-hydroxylated metabolite of Trp-P-2 (Fig. 7). One conjugate was a potent bacterial mutagen and assigned as the semimercaptal conjugate; detoxicated products were characterized as a GSH-linked sulfinamide, and a stable S-C conjugate formed at the C-4 atom of Trp-P-2 [173]. The GSTA and GSTP class isoforms of GST inhibited DNA binding of activated HONH-PhIP metabolites in cell-free systems. An incubation mixture containing *N*-acetoxy-PhIP, GSH, and GSTA1-1 produced oxidized GSH (GSSG) and reduced PhIP. GSH conjugates were not detected, suggesting that a nucleophilic displacement mechanism was involved in the deactivation of *N*-acetoxy-PhIP [174]. A short-lived GSH sulfenamide conjugate of PhIP may have formed and undergone a reaction with another GSH molecule to produce PhIP and GSSG (Fig. 7). GSTP-1 inhibited DNA binding of ATP-dependent metabolite(s) of HONH-PhIP [175]. The LNCaP human prostate adenocarcinoma cell line genetically modified to express GST P-1 also diminished HONH-PhIP DNA binding in intact cells [174, 175]. GSH depletion in primary rat hepatocytes by L-buthione-sulfoximine resulted in a 15-fold increase in PhIP-DNA adducts [176], and GSH depletion in vivo in rats resulted in a 5-fold increase in hepatic PhIP-DNA adducts [177]. The GST-dependent detoxication pathway may be a critical determinant for the organ specificity of PhIP-carcinogenesis in rodents and possibly humans [174, 178]. Human liver cytosol contains high levels of GST A1-1 that catalyzes the GST-mediated detoxication of *N*-acetoxy-PhIP; however, the colon cytosol contains about 100-fold lower levels of the GSTA1-1 and does not display GST-mediated inhibition of *N*-acetoxy-PhIP binding to DNA [174, 176, 179]. The high levels of hepatic GSTA1-1 expression may explain the lower levels of PhIP-DNA adduct formation in the liver compared to that in the pancreas, prostate, or colorectal tissue of rats which lack this enzyme [177]. Thus far, HAA mercapturic acid conjugates have not been identified in the urine of experimental laboratory animals or humans.

### HAA macromolecule adduct formation

#### HAA-DNA adducts

**Fig. 8:**
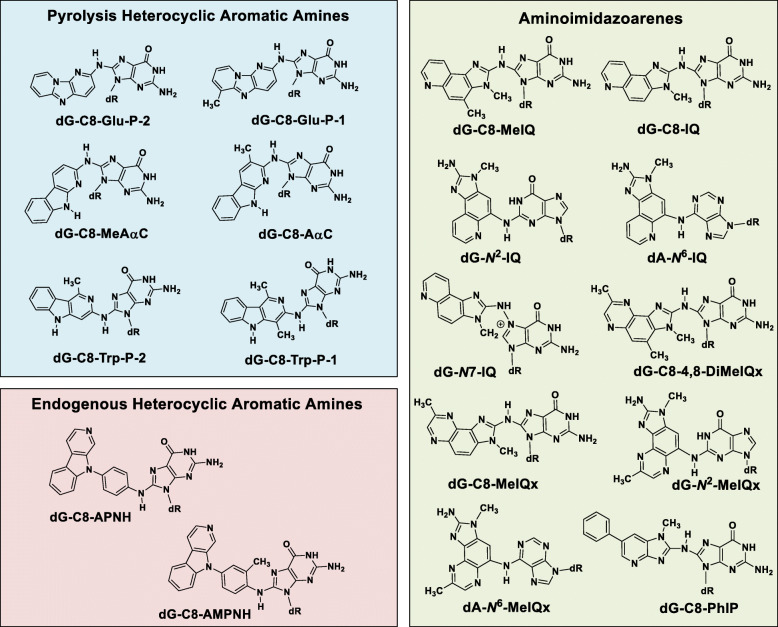
Structures of HAA-DNA adducts

**Fig. 9:**
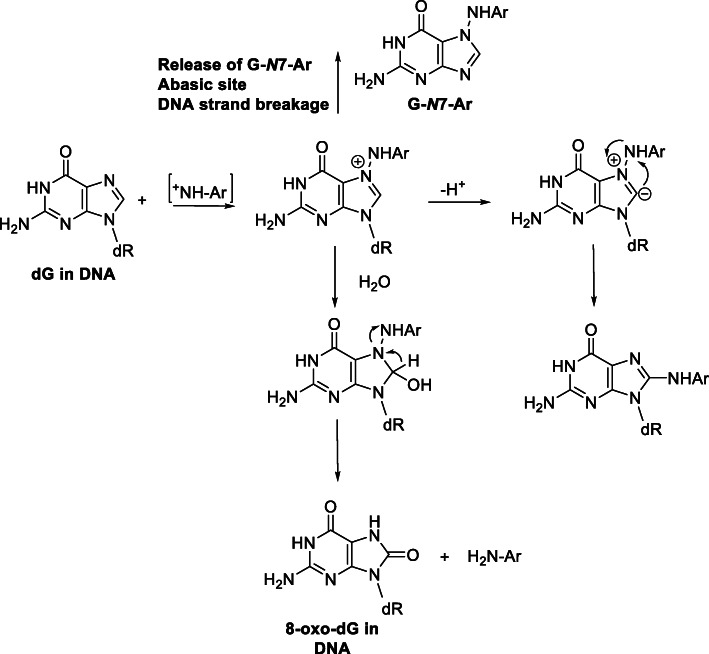
Proposed mechanism of dG-C8-Ar adduct formation, induction of abasic sites, DNA strand breakage, and 8-oxo-dG formation. Ar represents an aromatic amine or HAA

The structures of prominent HAA-DNA adducts are shown in Fig. 8. HAA-DNA adducts were originally prepared through biomimetic reactions of the HONH-HAA intermediates with deoxynucleosides or DNA with ketene gas, or acetic anhydride, to produce reactive *N*-acetoxy intermediates. The overall yield of DNA adduct formation with deoxynucleosides or DNA by biomimetic reactions is usually several percent or lower [49, 63, 152, 180–182]. *N*-Hydroxy-HAAs bind primarily to the C8 atom of dG. Minor reaction products of HONH-IQ and HONH-MeIQx also occur at the *N*^2^ atom of dG and the C8 and *N*^6^ atoms of dA [180, 182, 183]. However, the C8 position of dG is only weakly nucleophilic. Studies with structurally related aromatic amines proposed that dG-C8-arylamine adducts are rearrangement products of ylide intermediates of dG-*N*7-arylamine hydrazine adducts (Fig. 9) [184]. dG-C8-HAA adduct formation may occur through the same mechanism. In support of this chemistry, a proposed hydrazine-linked dG adduct of IQ, *N*^2^-(2′-deoxyguanosin-7-yl)-IQ (dG-*N*7-IQ) was identified and characterized by mass spectrometry [182] (Fig. 8). Based on studies with 4-nitroquinoline 1-oxide, cationic dG-*N*7-arylamine adducts also can undergo depurination to produce DNA strand breaks or undergo solvolysis to produce 8-oxo-2′-deoxyguanosine (8-oxo-dG) [185, 186]. These chemical mechanisms may explain dG-C8-HAA formation and HONH-HAA-mediated DNA strand breakage and 8-oxo-dG formation (Fig. 9) [187].

More recently, nonbiomimetic approaches using the Buchwald-Hartwig arylamination have been employed to synthesize multiple dG-C8-HAA adducts and 5-(2′-deoxyguanosin-*N*^2^-yl)-IQ (dG-*N*^2^-IQ) in larger scale [188–190]. Only several studies have investigated HAA-DNA structure and the fidelity of polymerases during translesional synthesis. An NMR study on the solution structure of dG-C8-PhIP situated within the oligonucleotide duplex d(CCATC**G**CTACC):d(GGTAGCGATGG) reported the PhIP adduct induced conformational exchanges in the DNA structure, existing between the major base-displaced intercalative structure and a minor external groove-binding structure [191, 192]. Similar conformational distortions were observed for DNA adducts of aromatic amines, which impact codon base pairing and implicated in mechanisms of mutagenesis [193]. Studies on the fidelity of several human polymerases during translesional syntheses across *N*-(2′-deoxyguanosin-8-yl)-PhIP (dG-C8-PhIP), *N*-(2′-deoxyguanosin-8-yl)-IQ (dG-C8-IQ), and 5-(2′-deoxyguanosin-*N*^2^-yl)-IQ (dG-*N*^2^-IQ) adducted oligomers showed the polymerase activities, and catalytic efficiencies were dependent on both DNA adduct structures and sequence contexts [194–197].

#### HAA-DNA adduct formation in experimental laboratory animals

HAA-DNA adducts have been detected in multiple tissues of experimental laboratory animals, employing ^32^P-postlabeling, liquid chromatography/mass spectrometry (LC/MS), or accelerator mass spectrometry (AMS), see citations in [49, 63, 198–201]. One study on DNA adduct persistence showed that dG-*N*^2^-IQ (Fig. 8), a minor DNA adduct formed in vitro with calf thymus DNA modified with *N*-acetoxy-IQ [180], becomes the prominent lesion in slowly dividing tissues of rats and nonhuman primates that underwent chronic treatment with IQ, indicating preferential repair of the major dG-C8-IQ adduct [202, 203]. Thus, the minor dG-*N*^2^-IQ may have a significant role in the tumorigenic properties of IQ.

#### HAA-DNA adduct formation in human tissues

The putative dG-C8-MeIQx adduct was detected in the colon and kidney of individuals at levels of several adducts per 10^9^ nucleotides when assayed by ^32^P-postlabeling [204]. dG-C8-APNH was detected in human lung and stomach tissue by LC/MS at levels ranging from 0.6 to 14 adducts per 10^8^ nucleotides [44]. A gas chromatography/negative ion chemical ionization-mass spectrometry assay, following the hydrolysis of presumed dG-C8-HAA adducts formed with DNA, was employed to measure adducts in DNA of the colorectal mucosa and lymphocytes of cancer subjects; the levels of the putative dG-C8-PhIP adduct were in the range of several adducts per 10^8^ DNA bases [205, 206]. ^32^P-Postlabeled lesions attributed to dG-C8-PhIP were detected in exfoliated breast epithelial cells in milk in 30 out of 64 lactating mothers [207]. The mean adduct level was 4.7 adducts/10^7^ nucleotides. In another study, PhIP-DNA adducts were detected by immunohistochemistry (IHC) in mammary tissue of 82% of the women with breast cancer (*N* = 106) and 71% of the tissue samples of the healthy control patients (*N* = 49) [208]. A very high percentage of pancreas and prostate biospecimens were also positive for PhIP-DNA adducts assayed by IHC [209, 210]. The detection limit of PhIP-DNA adducts by IHC is several adducts per 10^7^ nucleotides, implying most patients harbored PhIP-DNA adducts at relatively high levels.

The frequent detection of PhIP-DNA adducts at high levels in human tissues is alarming and implies that PhIP-DNA adduct formation occurs with far greater efficiency in humans exposed to ppb levels of dietary PhIP than in rodents given high, carcinogenic doses of PhIP (10 - 50 mg/kg body weight). However, the biomarker data obtained by ^32^P-postlabeling and IHC are controversial: both screening methods are non-selective and fail to provide confirmatory spectral data, and thus, the identities of the lesions are equivocal. PhIP-DNA adducts measured by AMS in the breast tissue of female cancer patients who had received a dose of [^14^C]PhIP (20 μg PhIP/70 kg body weight) by oral administration before surgery ranged from 26 to 480 adducts/10^12^ nucleotides [211], or nearly 1000- to 10,000-fold lower than the levels of adducts reported by the IHC or ^32^P-postlabeling techniques [207, 208]. Our laboratory employed a specific and sensitive LC/MS ion trap method to screen non-tumor-adjacent mammary tissue for DNA adducts of PhIP [212]. Only 1 of 70 biopsy samples obtained from breast cancer patients from Minneapolis harbored the dG-C8-PhIP adduct. The level was three adducts per 10^9^ nucleotides, a level that is 100-fold lower than the mean level of PhIP adducts reported in other cohorts monitored by IHC or ^32^P-postlabeling methods. Our laboratory also reported a much lower detection frequency and lower adduct levels of dG-C8-PhIP in human prostate specimens assayed by LC/MS than the IHC assays [213, 214].

**Fig. 10:**
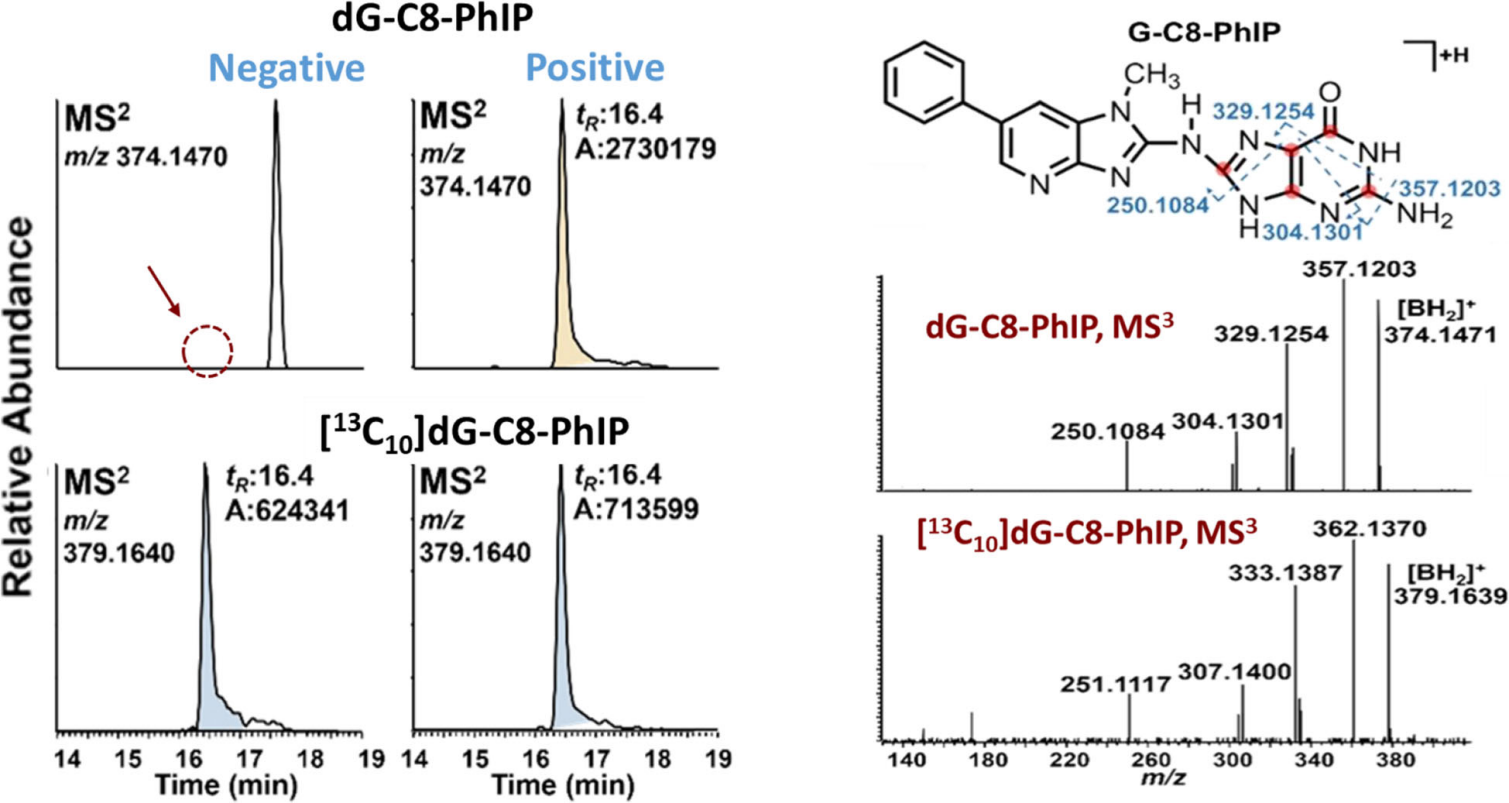
Reconstructed mass chromatograms at the MS^2^ scan stage of a human prostate sample targeting dG-C8-PhIP. One subject is shown with dG-C8-PhIP below the detection limit, and a second patient is positive for dG-C8-PhIP. [^13^C_10_]-dG-C8-PhIP was employed as the internal standard at a level of 3 adducts per 10^8^ nucleotides. The MS^3^ scan stage product in spectra confirmed the identities of dG-C8-PhIP and its internal standard. Proposed MS^3^ fragmentation pathways are displayed; isotopically labeled ^13^C atoms of the internal standard are marked in red. Adapted with permission from [214]

HAA-DNA adduct levels and frequency of detection may significantly differ between cohorts and are dependent on the cohort's dietary preferences for consuming well-done cooked meats and the analytical methods employed to measure DNA adducts. The IHC assay detected PhIP-DNA adducts at high frequency, occurring at levels at or above several adducts per 10^7^ nucleotides in PC patients treated at the Henry Ford Health System in Detroit, MI [210, 215]. In contrast, our method employing high-resolution accurate mass spectrometry reported a significantly lower detection frequency of dG-C8-PhIP (13 out of 164 patients) in PC patients treated at the Department of Urology, University of Minnesota Medical School, Minneapolis, MN (Fig. 10) [213, 214, 216]. The dG-C8-PhIP levels ranged between 0.3 to 12 adducts per 10^8^ nucleotides. DNA adducts of other prevalent HAAs were not detected [214]. The large discrepancy in detection frequency and PhIP adduct levels in the prostate (and breast) obtained by IHC and ^32^P-postlabeling, as opposed to the precise AMS or LC/MS methods, suggests that the biochemical assays are non-specific and detect a variety of lesions in addition to or other than dG-C8-PhIP. These findings call for further studies, where DNA adduct levels detected by biochemical and MS methods are compared directly for the same tissue samples. We believe that such direct comparisons will demonstrate the necessity to employ specific MS methods to measure DNA adducts in human population studies.

#### HAA blood protein adduct formation

Carcinogen adducts with hemoglobin (Hb) and serum albumin (SA) have been characterized for many classes of carcinogens, including aromatic amines, HAAs, polycyclic aromatic hydrocarbons (PAHs), AFB_1_, and other alkylating agents [49, 217–223]. The Hb lifetime is 125 days, and the half-life of SA is 20 – 25 days in humans [217, 224]. Thus, the steady-state levels of stable Hb and SA carcinogen adducts may reach, respectively, about 60- and 29-fold higher after chronic exposure than after a single dose. Several blood protein carcinogen adducts have served as long-lived biomarkers in human studies [49, 217–223].

#### HAA hemoglobin adducts

**Fig. 11:**
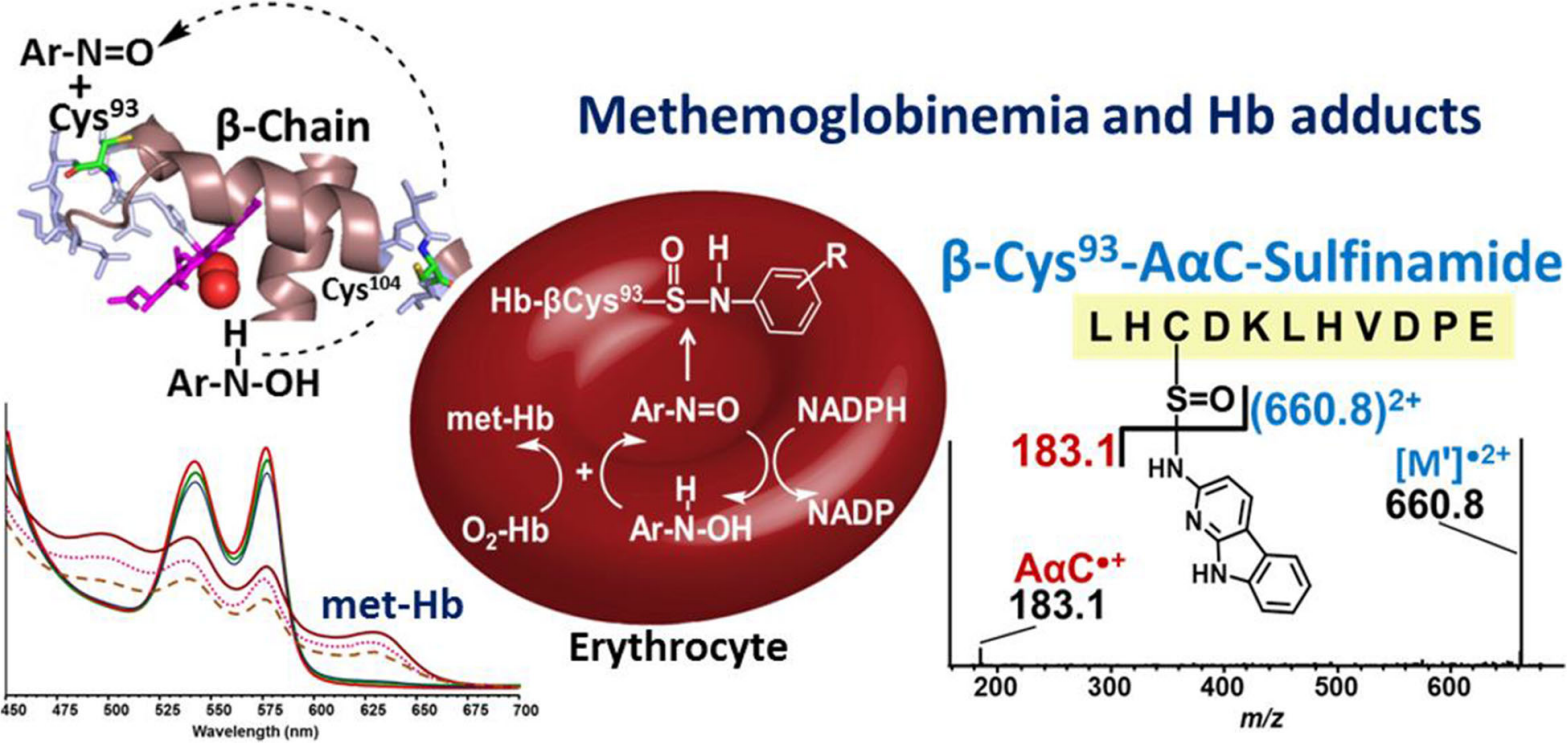
Co-oxidation of HbO_2_ by N-hydroxylated aromatic amines and HAAs in human RBCs and met-Hb formation. The mass spectrum of the AαC sulfinamide formed at the β-Cys^93^ of Hb was acquired by ion trap mass spectrometry employing electrospray ionization in the positive ion mode. The AαC-modified Hb was digested with Glu-C, and the peptide sequence encompassing the AαC sulfinamide adduct formed at Hb β-Cys^93^ is reported. Adapted with permission from [226]

Structurally related arylamines undergo metabolism by hepatic cytochrome P450 to form the arylhydroxylamine metabolites, which undergo systemic circulation through the blood [225]. Once taken up by the erythrocytes, the arylhydroxylamines can undergo a co-oxidation reaction with oxy-hemoglobin (HbO_2_) to form methemoglobin (met-Hb) and the arylnitroso intermediates. Arylnitroso compounds can undergo redox cycling with NADPH reductase in the erythrocyte to reform the arylhydroxylamines, ultimately producing methemoglobinemia (Fig. 11). Some arylnitroso intermediates also selectively react with Hb β-Cys^93^ to form Hb arylsulfinamide adducts. The biochemistry of arylamine metabolism has been exploited to biomonitor several AAs, including aniline (ANL), *o*-toluidine, 2-naphthylamine, and 4-aminophenyl (4-ABP) through their Hb arylsulfinamide adducts in humans [227, 228]. In contrast to aromatic amines, most HAAs studied do not efficiently bind to rodent or human Hb in vivo [49, 201, 229, 230]. We compared the reactivity of the synthetic *N*-hydroxy metabolites of PhIP, MeIQx, AαC, ANL, and 4-ABP with purified human HbO_2_. Phenylhydroxylamine (HONH-ANL), *N*-hydroxy-4-aminobiphenyl (HONH-4-ABP), and HONH-AαC rapidly oxidized HbO_2_ to met-Hb, forming arylamine and AαC sulfinamide adducts with a concomitant decrease in the free Hb β-Cys^93^ thiol content of Hb [226]. In contrast, HONH-PhIP and HONH-MeIQx did not induce oxidation of HbO_2_, the number of titratable thiols remained unchanged from the control, and chemical modification of the Hb β-Cys^93^ with either carcinogen was negligible. Like HONH-ANL and HONH-4-ABP, HONH-AαC underwent co-oxidation with HbO_2_ in isolated human erythrocytes to produce methemoglobinemia, while HONH-PhIP and HONH-MeIQx did not (Fig. 11). Molecular modeling studies showed that the HONH-AA or HONH-HAA substrates were too distant to interact with HbO_2_ and form met-Hb. The different conformational changes in flexible helical and loop regions around the heme pocket induced by the HONH-AAs or HONH-HAAs may explain the different proclivities of these chemicals to induce methemoglobinemia [226]. HONH-AαC formed the Hb sulfinamide adduct in erythrocytes at greater levels than HONH-4-ABP, suggesting that the AαC Hb sulfinamide may be a promising biomarker to assess AαC exposure in humans [226].

AαC is present in mainstream tobacco smoke at 25 − 100-fold higher amounts than 4-ABP [23, 231–233]; however, the mean level of the 4-ABP-Hb adduct is 7.4-fold greater than the mean level of the AαC-Hb adduct in smokers (Table 4) [234]. The higher level of 4-ABP-Hb adduct formation is not attributed to the superior reactivity of HONH-4-ABP with HbO_2_ since HNOH-AαC induces more methemoglobinemia in erythrocytes in vitro and forms higher levels of the Hb-sulfinamide than does HONH-4-ABP [226]. Higher 4-ABP N-oxidation rates do not appear to explain the elevated levels of 4-ABP-Hb formation as the hepatic microsomal N-oxidation rates of both compounds are similar [108, 235]. AαC undergoes extensive metabolism by primary human hepatocytes to produce multiple phase I and phase II metabolites [130], whereas the pathways of metabolism of 4-ABP in human hepatocytes are incompletely characterized [236]. We postulate that the amount of HONH-4-ABP circulating in the blood and delivered to the erythrocyte is far greater than HONH-AαC, resulting in proportionately higher levels of 4-ABP-Hb adducts [228, 237]. Neither 4-ABP nor AαC DNA adducts were detected in white blood cells of these smokers (limit of quantification 3 adducts per 10^9^ nucleotides) [234].

**Table 4:** Hb adducts of 4-ABP, AαC, cotinine levels, and smoking status^a^

	4-ABP-Hb (pg/g Hb)	AαC-Hb (pg/g Hb)	Cotinine (ng/mL)	Cigarettes per day	Smoking years
Commercial Blood	22.9 ± 0.2b^b^	4.3 ± 2.4	n.a^c^	n.a	n.a
R01	29.8 ± 6.9	5.6 ± 0.8	0.13	0	0
R03	23.8 ± 6.9	4.6 ± 0.6	0.13	0	0
R05	21.8 ± 2.8	5.6 ± 1.1	0.13	0	0
R02	189 ± 8.5	16.2 ± 1.9	164	30	42
R04	71.4 ± 8.9	16.1 ± 1.0	224	20	25
R06	125 ± 6.8	26.8 ± 2.5	228	21	33
R07	134 ± 7.5	24.5 ± 0.8	210	30	32
R08	71.3 ± 1.6	17.3 ± 0.7	86	25	30
R09	162 ± 7.8	15.5 ± 0.9	108	70	53
R10	90.4 ± 3.7	6.6 ± 0.6	55	25	30

^a^reproduced with permission from [234]

^b^mean ± standard deviation

^c^n.a., not analyzed

#### HAA serum albumin adducts

SA is the most abundant serum protein in humans. It is synthesized in the hepatocyte and released from the endoplasmic reticulum as a mature protein with 585 amino acids after cleavage of the signal peptide sequence [238]. Albumin is critical in maintaining the colloid osmotic pressure and transporting hormones, fatty acids, and xenobiotics, including therapeutic drugs, environmental pollutants, and carcinogens [239]. Several classes of carcinogens, including many HAAs and AFB_1_, react poorly with Hb [201, 229, 230, 240], possibly because the reactive intermediates do not escape from the hepatocyte. However, SA carcinogen adducts can form in the hepatocyte, where metabolic activation occurs for many genotoxicants, including HAAs and AFB_1,_ [64, 108, 240, 241]. Thus, monitoring SA adducts of certain carcinogens may be feasible when they fail to bind to Hb.

The Cys^34^ is one of 35 conserved cysteine residues in SA across species [239]. Thirty-four of these cysteines are involved in 17 disulfide bonds. The single unpaired Cys^34^ is present either as a free thiol or in an oxidized form as mixed disulfide linkages with low molecular weight thiols [242–244]. The SA-Cys^34^ resides in a crevice in a microenvironment close to three ionizable residues, Asp^38^, His^39^, and Tyr^84^, resulting in an unusually low pKa value of ∼6.5 for the Cys^34^ thiol compared to pKa values of about 8.0 − 8.5 for Cys thiols in many other proteins and explain the high reactivity of SA-Cys^34^ [245–248]. The scavenging properties of the SA-Cys^34^ with xenobiotic electrophiles are well documented [220, 221]. N-Oxidized metabolites of MeIQx, PhIP, and IQ form sulfenamide or sulfinamide adducts at the Cys^34^ of rodent or human SA [230, 249–251].

**Fig. 12:**
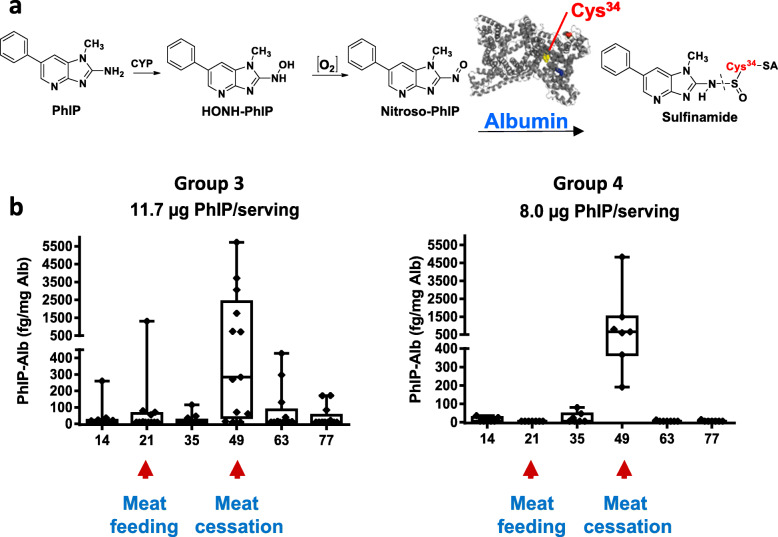
**a** Mechanism of acid-labile PhIP sulfinamide adduct formation at SA-Cys^34^. **b** Kinetics of formation and removal of this acid-labile PhIP-SA adduct in plasma of subjects on a cooked meat diet containing known quantities of PhIP. Box and whiskers plot showing the distribution of PhIP-SA measured over the entire study, separately for Group 3 (*N* = 13) and Group 4 (*N* = 7) ingested PhIP during the meat-feeding phase (days 22–49). Adapted with permission from [252]

The SA-Cys^34^ is a primary binding site of HONH-PhIP in vitro and in vivo in humans, occurring as a sulfinamide (Fig. 12a) [253–256]. We investigated the kinetics of PhIP-SA adduct formation in volunteers on a semi-controlled meat feeding study for 4 weeks (Fig. 12b) [252]. PhIP-SA adduct levels were low during the three-week pre-feeding phase but increased during the four-week meat diet. Thus, PhIP-SA adduct formation proves PhIP underwent bioactivation in vivo. However, the PhIP-SA adduct declined rapidly, decreasing by 96% two weeks after completing the meat diet, signifying that the PhIP-Cys adduct is unstable and probably unsuitable for biomonitoring subjects on free-choice diets. The PhIP-SA adduct levels varied over 550-fold among the subjects after four weeks of a meat diet; in contrast, the urinary Gluc metabolites of HONH-PhIP in these subjects only varied by 2-fold [213, 257]. Correlations were not seen between PhIP-SA adduct levels and the amount of PhIP accrued in hair (*P* = 0.13), the amounts of N-oxidized urinary metabolites of PhIP (*P* = 0.66), or caffeine CYP1A2 activity (*P* = 0.55), a key enzyme involved in PhIP metabolism [252]. PhIP has multiple metabolic fates that can obscure the relationship between urinary HONH-PhIP-Gluc levels and PhIP-SA adducts [49]. The large intraindividual variations of CYP1A2 phenotype and inconsistent responses to CYP1A2 inducers also obscured the relationship between CYP1A2 phenotype and PhIP-SA adduct levels [257].

**Fig. 13:**
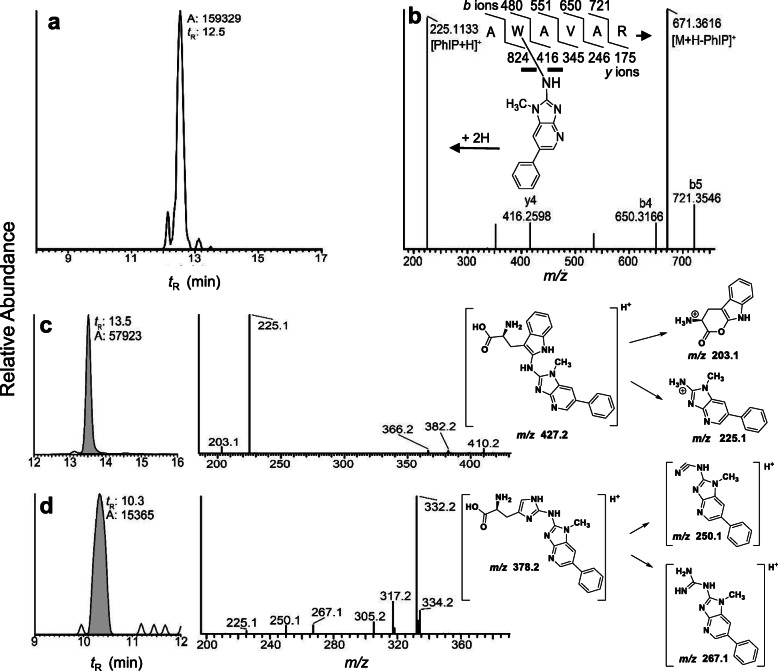
*N*-Acetoxy-PhIP modified human SA. Reconstructed mass chromatograms of **a** AW*[PhIP] AVAR ([M + 2H]^2+^ *m/z* 448.2385 > *m/z* 225.1135, *m/z* 671.3624; mass tolerance 2 ppm), **b** MS^2^ product ion spectrum of AW*[PhIP] AVAR ([M + 2H]^2+^, **c** Product ion spectrum of W*PhIP adduct following digestion with Pronase E, and **d** Product ion ion spectrum of the H*PhIP adduct following digestion with Pronase E and proposed structures. The base peak at m/z 332.2 is attributed to the loss of formic acid. Adapted with permission from [255]

PhIP adducts also formed at the sole Trp^214^ of SA in human plasma treated in vitro with *N*-acetoxy-PhIP [255], recovered as AW*[PhIP] or W*[PhIP], respectively, in tryptic or pronase digests (Fig. 13). Another adduct was detected at His(s) following pronase digestion [255]. Trp and His adducts form at ~ 10-fold lower levels than PhIP-SA-Cys^34,^ but these adducts are stable linkages and may accumulate during chronic exposure to PhIP and prove pivotal as long-term biomarkers in biomonitoring of PhIP.

#### Biomonitoring HAAs in hair

**Fig. 14:**
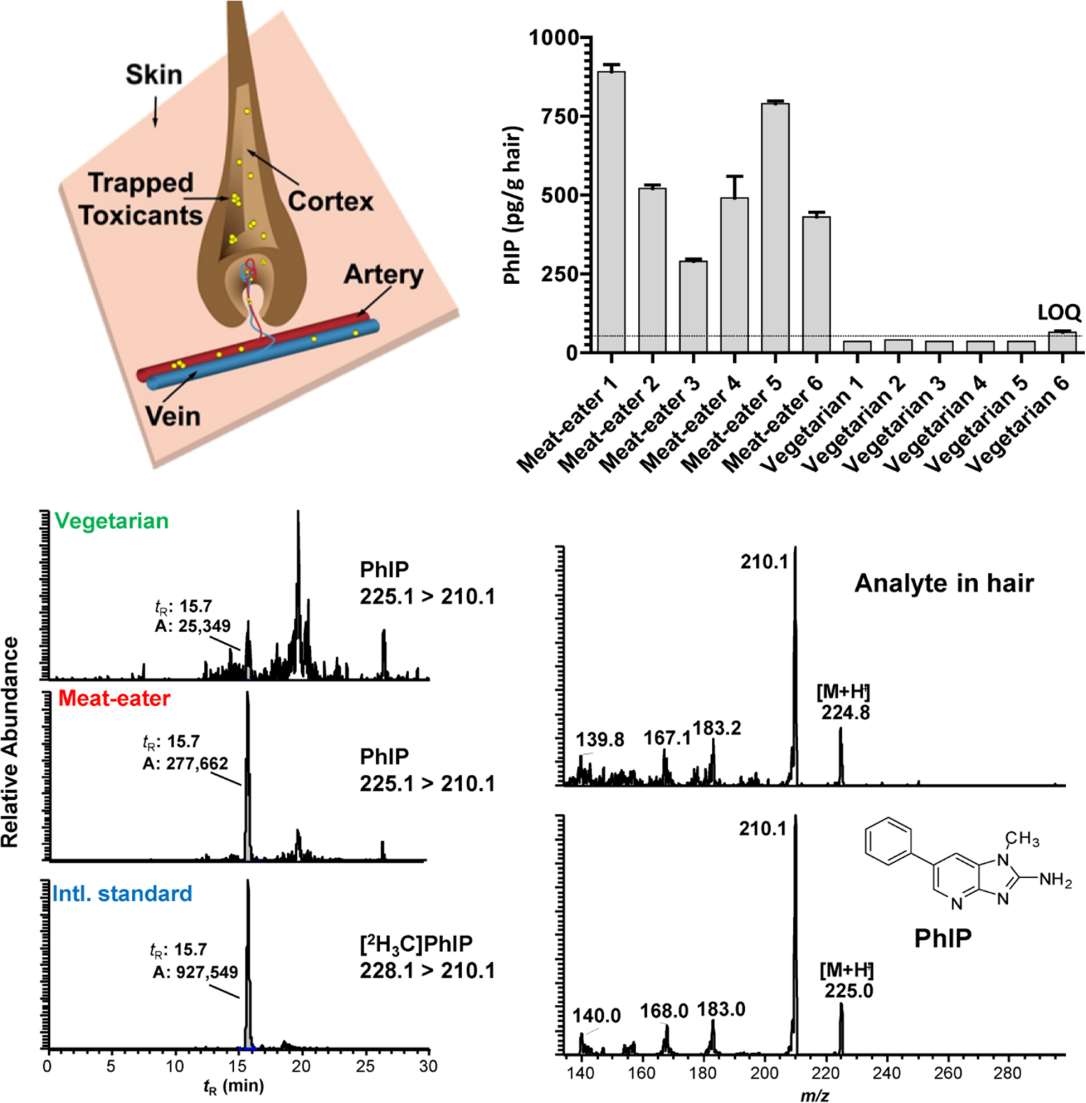
Measurement of PhIP in the hair of omnivores and vegetarians. The full scan product ion spectrum acquired by triple quadrupole mass spectrometry confirmed the identity of PhIP. Adapted with permission from [263]

**Fig. 15:**
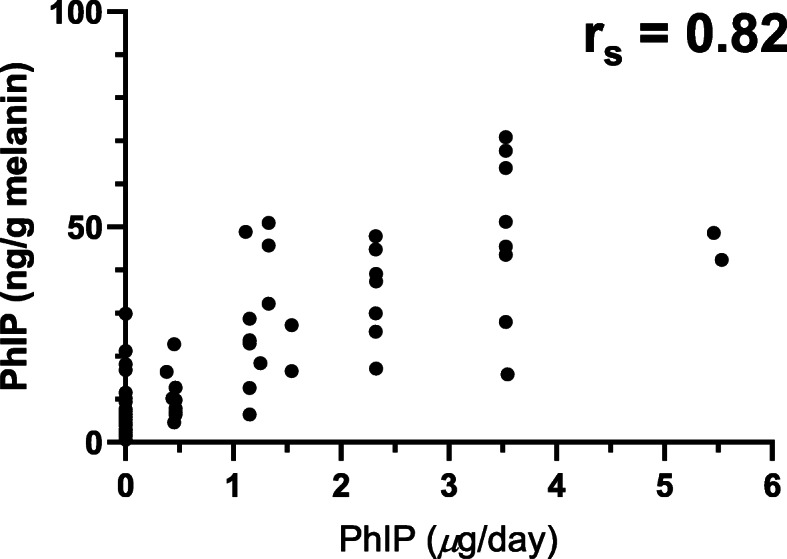
Correlation of post-feeding PhIP scalp hair levels normalized for melanin in healthy volunteers with variable dietary PhIP intake 5 days a week over 4 weeks. The 0 value represents PhIP hair levels before commencing the feeding study. Adapted with permission from [268]

Stable, long-lived biomarkers are still sought to improve exposure assessment of HAAs than the currently used FFQ [258, 259]. Human hair has served as matrices for biomonitoring a wide range of chemicals, including nicotine, drugs, and narcotics, and hormones [260–262]. Our laboratory and others have shown that once PhIP is ingested, a small portion of the dose becomes entrapped in the hair follicle and incorporated into the newly grown hair shaft (Fig. 14) [259, 263, 264]. The degree of PhIP binding to hair is strongly correlated to the eumelanin content [265, 266]. In contrast, other prevalent HAAs are sequestered in human hair at much lower levels. We established a tandem solid-phase extraction method followed by triple quadrupole mass spectrometry to quantify PhIP in hair, which requires only 25 mg of hair [263, 267, 268]. PhIP was detected in the hair of omnivores but not vegetarians, demonstrating PhIP exposure is derived from a cooked meat diet (Fig. 14). Thereafter, we measured PhIP in the hair of nonsmokers who completed a semi-controlled diet of ground beef cooked to different doneness levels, reflecting a low or high concentration of PhIP in the range found in the American diet for 5 days a week for 1 month [268, 269]. Newly grown hair clipped from the back of the subjects’ heads near the nape of the neck captured the previous 1-month exposure of PhIP. A substantial increase in PhIP hair levels occurred as a function of dose and normalizing for melanin content strengthened the correlation (ρ = 0.68, *P* < 0.0001) (Fig. 15). However, CYP1A2 activity, based on the urinary caffeine metabolic ratio, had no impact on PhIP hair levels [267, 268]. The intraindividual CYP1A2 activity was highly variable over the time course of the feeding study and precluded stratification of subjects’ CYP1A2 phenotype activity. PhIP hair levels may serve as a biomarker of exposure in epidemiologic studies investigating the association of cooked meat, HAAs, and cancer risk.

#### Is PhIP a human prostate carcinogen?

PC is the second most common cancer in men worldwide, with an estimated 1.1 million identified cases and 0.3 million PC-related deaths occurring by 2012 [270]. Studies of migrant populations show a significant increase in PC incidence and mortality rates in migrants from low prevalence PC regions following their relocation to a high prevalence of PC countries [271, 272]. Thus, environmental and/or dietary factors are involved in PC development. Several dietary components, including high-fat, dairy products, alcohol, and red meats, are associated with PC risk [273], although the specific dietary compounds and their mechanisms of action remain undefined. Other PC risk factors include; age, family history, and ethnicity, with African-American (AA) men having a two-time higher risk than Caucasians [274].

The majority of epidemiologic studies investigating dietary consumption of well-done cooked meat in relation to various tumor sites trend to a positive association between cancer risk and well-done meat consumption [87, 88, 275, 276]. Although, the data are inconsistent. A review and meta-analysis concluded that red meat intake was not linked to PC risk [277]; however, subgroup analyses revealed that frequent intake of well-done grilled or barbequed red meat was linked to aggressive PC [89, 278–281]. Thus, a health risk for PC may occur primarily by consuming well-done grilled or barbequed meats containing elevated levels of genotoxicants, including HAAs and PAHs [2, 282]. HAAs and PAHs are cancer initiators [2, 49, 283, 284], and some may serve as tumor promoters [278, 285, 286]. PhIP is the only cooked mutagen reported to induce PC in rodents [2, 13, 90]. The rodent carcinogenicity studies were conducted at doses of PhIP that are more than a million-fold higher than its daily intake in humans [2]. However, linear-dose relationships exist between PhIP, MeIQx, and IQ, and DNA adduct formation in rodents down to human exposure levels, and DNA adducts of PhIP and MeIQx have been unambiguously identified in several human organs when assayed by specific AMS [201, 211, 287, 288], or LC/MS methods for dG-C8-PhIP in the prostate (Fig. 10).

The mortality rate of PC in AA men is twice as high as white men in the United States [289]. The elevated PC risk is due to genetic factors, and exposures to hazardous chemicals in the environment and diet also may contribute to PC. AA men reportedly eat more well-done cooked meats and poultry containing PhIP than Caucasian men [269, 290]. One study reported elevated levels of the prostate-specific antigen (PSA), a biomarker of PC risk, was associated with dietary PhIP intake in a prospective clinic-based study among AA men [289]. Does PhIP play a role in PC development, or is a combination of the complex panel of chemicals present in cooked meat contributing to increased aggressive PC risk for AA men? The role of the well-done cooked meat diet in PC risk among different ethnic groups requires further investigation.

#### PhIP genotoxicity in human prostate cells

**Fig. 16:**
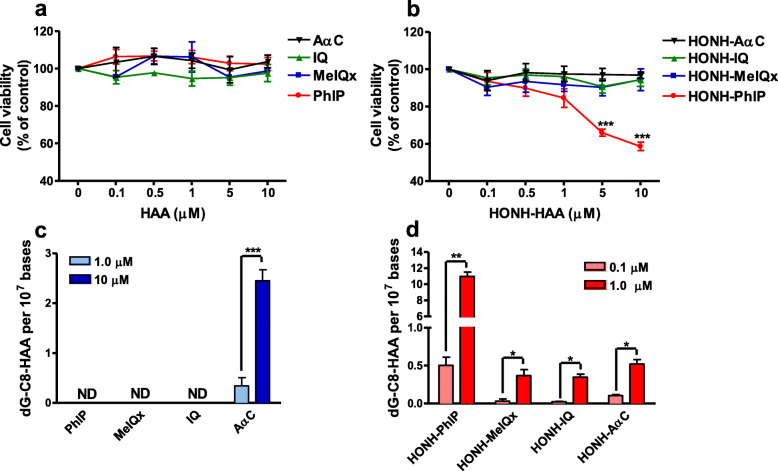
HAA and HONH-HAA cytotoxicity and DNA adduct formation in LNCaP cells. **a** and **b** LNCaP cells were treated with DMSO (0.1%), HAAs, and HONH-HAAs (0.1 μM – 10 μM), for 24 h. Cell viability was evaluated by the MTS assay. **c** and **d** HAA and HONH-HAA DNA adduct formation in LNCaP cells after 24 h were measured by UPLC-ESI/MS^3^. Data are representative of at least three different experiment and are expressed as mean ± SD. ND: not detected. Ctrl: control

**Fig. 17:**
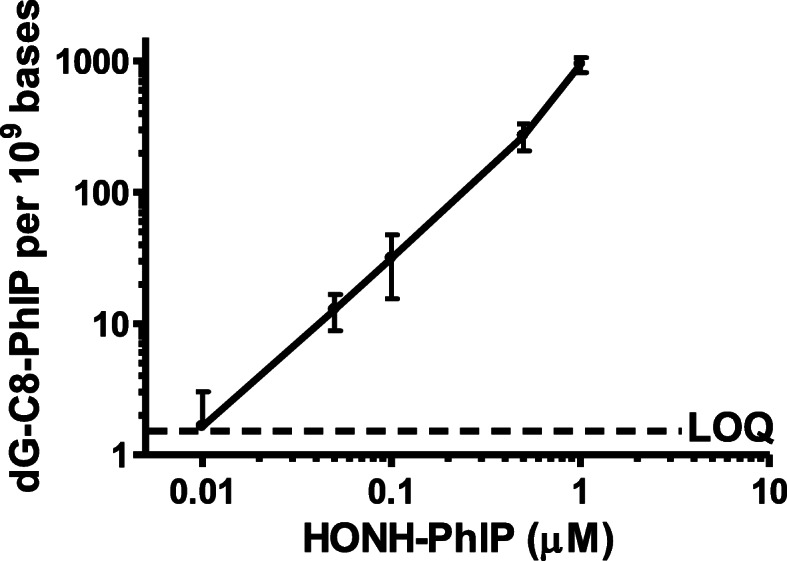
dG-C8-PhIP DNA adduct formation as a function of HONH-PhIP (10 nM – 1000 nM) dose in LNCaP cells treated for 8 h. dG-C8-PhIP was measured by UPLC-ESI/MS^3^. Data are representative of at least three different experiments and are expressed as mean ± SD. LOQ: limit of quantification

Our and other laboratories have studied HAA-DNA adduct formation and cytotoxicity in cancerous human prostate cell lines and primary prostate epithelial cells [175, 213, 291, 292]. We employed LNCaP cells, an androgen-sensitive prostate adenocarcinoma cell line derived from supraclavicular lymph node metastasis, for studies with HAAs. PhIP, MeIQx, and IQ did not form DNA adducts and were not toxic because the CYP1 enzymes, which bioactivate HAAs, are poorly expressed in LNCaP cells [213]. AαC did form DNA adducts at low levels probably through bioactivation with other CYP or non-CYP oxidases expressed in LNCaP cells [113, 213] (Fig. 16). These data indicate that the CYP mediated N-oxidation step of most HAAs likely occurs in the liver, followed by the systemic circulation of the HONH-HAAs to the prostate, where further bioactivation by Phase II enzymes can occur [213]. The synthetic HONH-MeIQx, HONH-AαC, and HONH-IQ intermediates were not cytotoxic at a dose up to 10 μM, but HONH-PhIP induced a concentration- and time-dependent cytotoxicity in LNCaP cells [213]. HONH-PhIP underwent efficient bioactivation by NATs and SULTs, leading to DNA adduct formation at levels that were 20-fold higher than the other HONH-HAAs in LNCaP cells [213]. PhIP-DNA adducts formed in a dose-dependent manner down to levels of human exposure (Fig. 17) [213]. Similarly, HONH-PhIP formed DNA adducts at levels that were up to 100-fold higher than those formed by HONH-MeIQx in normal prostate epithelial cells from the transition zone [291, 292]. HONH-PhIP also induced unscheduled DNA synthesis and DNA single-strand breaks in primary human prostate epithelial cells at levels that were up to 100-fold higher than HONH-MeIQx [293, 294].

dG-C8-PhIP, the sole known adduct formed with PhIP [152, 181, 295], induces CG>AT transversions followed by CG>TA and CG>GC transitions in rodents, transgenic target genes, and site-specific mutagenesis studies [62, 194, 296, 297]. The SBS8 mutation signature extracted from prostate adenocarcinomas reported in the Pan-Cancer Analysis of Whole Genomes (PCAWG) is of unknown etiology with transcriptional strand bias on CG>AT, suggesting mutations were induced by guanine adducts; the SBS18 mutational signature is linked to oxidative stress; SBS33 is of unknown etiology with strand bias on TA>CG, suggestive of thymidines adducts; and SBS37 and SBS41 are of unknown etiology [298]. Recently, a CG>AT pattern of PhIP-induced mutations characterized in human induced pluripotent stem cells share 60% similarity with the oxidative-stress-related CG > AT background mutation pattern [299]. Other components in red meat, such as heme iron, can damage organs by inducing free radical formation, and lipid peroxidation products formed in cooked meat also may form DNA adducts or serve as tumor promoters [286]. Studies on the role of PhIP, other cooked meat genotoxicants and meat-related chemicals in DNA adduct formation, mutational signatures, and DNA repair in human prostate epithelial cells are warranted to advance the mechanisms by which chemicals in cooked meat contribute to prostate mutagenesis.

In addition to its tumor-initiating activity, PhIP may act via non-genotoxic mechanisms, possibly through the AR, leading to cell proliferation, inflammation, and PC development [90, 97, 98, 300]. Molecular docking simulation showed that PhIP and HONH-PhIP bound to the AR at comparable affinities to those of the endogenous AR ligand, dihydrotestosterone (DHT), and their binding competes with DHT in the native AR binding cavity of the receptor [300]. Moreover, LNCaP treatment with PhIP or HONH-PhIP resulted in the up-regulation of the expression of both AR and PSA protein, demonstrating that both PhIP compounds activate AR [300]. In addition, PhIP at nM concentrations also induced proliferation, migration, and invasion in PC-3, an AR negative human prostate cell line, resulting from the ERK signal transduction cascade [301]. Thus, PhIP may act through androgenic mechanisms and other signal transduction pathways to induce or promote PC at physiologically relevant concentrations.

#### PhIP metabolomics in prostate cells

**Fig. 18:**
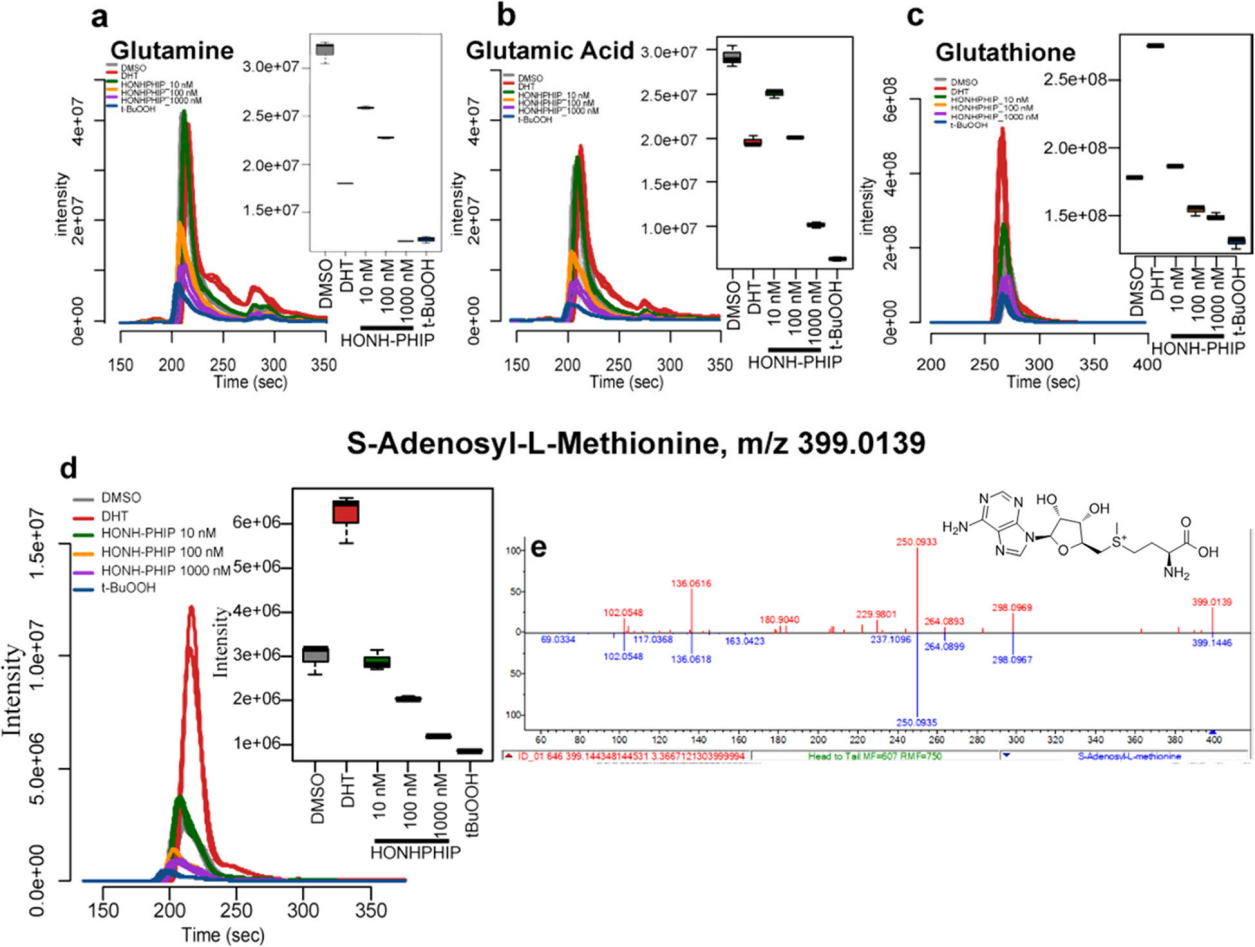
LNCaP cells (1 million cells) were incubated with test compounds HNOH-PhIP (10, 100 or 100 nM), DHT (1 nM), or *t*-BuOOH (500 μM) for 24 h [302]. Cells were lysed with 80% methanol/20% water containing 0.2% formic acid and extracts, concentrated by vacuum centrifugation, and analyzed by LC/MS with the Orbitrap Lumos at 120 K resolution [302]. Extracted ion chromatograms (5 ppm tolerance) for selected metabolite precursor ions and box and whisker plots depicting altered profiles for **a** glutamine, **b** glutamic acid, **c** GSH, **d** SAM, and **e** the product ion spectrum and the match to NIST 2019 mass spectrometry small molecule library entry supports the the identity of SAM

**Fig. 19:**
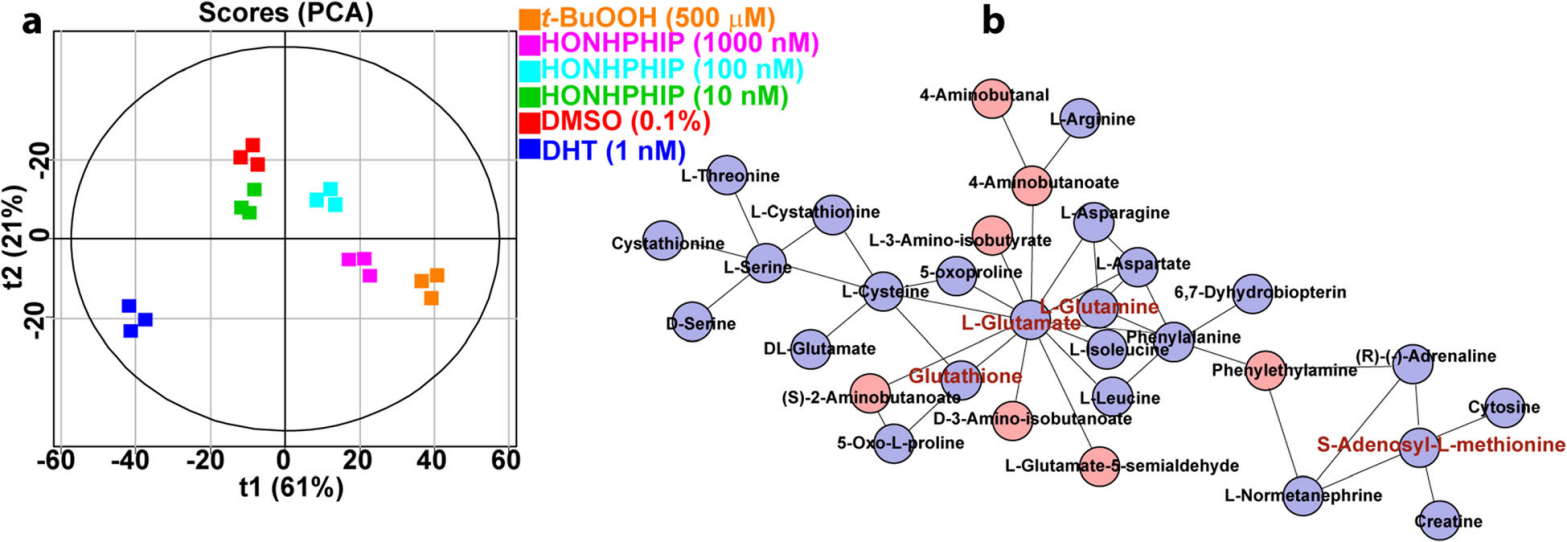
PCA plots and molecular networks of LNCaP cells following treatment with HONH-PhIP (10, 100 or 1000 nM), DHT (1 nM), or *t-*BuOOH (500 μM). Data were processed using MetaboAnalyst 3.0 [309], R3.5 for statistics, and *mummichog* for molecular ID and pathways analysis [308]. **a** Statistical clustering result using *ropls* R package for the HRMS metabolite intensities showing the separation of the treatment groups. **b** *mummichog* molecular network of detected metabolite masses contributing toward the enrichment of multiple metabolomic pathways. The network of nodes and lines are molecules from metabolic networks detected using high-resolution accurate *m/z* values and *mummichog* with a database of possible *m/z* values and ion types for metabolites in humans. Round, colored nodes each represent a metabolite ion detected in the experiment. Pink and blue nodes depict molecules that increased or decreased (*p* < 0.01), respectively, in the HONH-PhIP-treated cells compared to the DMSO control. Lines indicate connections between metabolites within the metabolic network. Red labeled metabolites are profiled by LC/MS^2^ in Fig. 18

PhIP induces molecular changes in AR and other proteins involved in PC in vivo in rodent prostate that can impact the metabolome [90]. We examined the effects of HONH-PhIP (0, 10, up to 1000 nM) on the LNCaP metabolome, [302] employing DHT and tert-butyl hydroperoxide (*t*-BuOOH) as test compounds for cell proliferation [303] and oxidative stress [304], respectively, since PhIP induces both biological events in the rodent prostate [98]. Statistical clustering of metabolite intensities produced distinct separations between the control and HONH-PhIP, DHT, and *t*-BuOOH treated cells indicating changes in the metabolic profiles following a 24-h treatment. Decreased glutamate and glutamine levels, intermediates in GSH biosynthesis, and decreased levels of GSH were detected. GSH is the most abundant antioxidant in the cell and the major player in cytoprotection against oxidative stress, whereas amino acid biosynthesis is linked to AR activation [305]. Oxidative stress and AR activation are critical events for PC development [303, 306]. S-Adenosyl-L-methionine (SAM) levels, an intermediate in GSH biosynthesis, and a key methyl donor that modulates DNA methylation and gene expression [307], also decreased in cells treated with HONH-PhIP and *t*-BuOOH but increased in DHT-treated cells (Fig. 18). Molecular identification followed by *mummichog* pathway and network analysis probed the metabolites and metabolic pathways altered by these chemical treatments [308]. The analyses were guided by preselection of significantly altered metabolites (ANOVA and Tukey HSD, *p* < 0.01), which identified several molecules in 13 metabolic pathways that were significantly up-or down-regulated by HONH-PhIP (Fig. 19). This data implies that HOHN-PhIP can serve as a modulator of gene and protein activity, impacting the metabolome [310]. However, LNCaP cells harbors mutations, including the loss of UGT and GST expression, two Phase II enzymes involved in HAA detoxication [213, 311], and contain multiple deleterious mutations in DNA repair genes, which impact the biological effects of HONH-PhIP [312]. Thus, our preliminary studies in LNCaP cells should be interpreted cautiously and may not accurately portray the metabolomic changes, DNA damage, and mutations induced by HONH-PhIP in healthy prostate cells. Mechanistic studies in normal primary prostate epithelial cells are required to advance our knowledge on the role of PhIP and its metabolites in prostate cancer biology.

## Conclusions

More than 20 HAAs form in high-temperature cooked red and processed meats, poultry, fish, and some HAAs arise in combusted tobacco. HAAs are mutagenic in bacterial and mammalian cells, and they induce mutations in oncogenes and tumor suppressor genes of rodents fed HAAs as part of the diet. HAAs induce cancers in the colon, pancreas, breast, and prostate of rodents; these are common cancer sites in Western countries where meat consumption is prevalent. Thus, there is much interest in the potential role of HAAs in meat-associated cancers. The mutational signatures of PhIP show similarity to COSMIC SBS4, SBS18, and SBS29 mutational signatures found in several types of human tumors [65]. However, these signatures are not unique to PhIP, and DNA adducts of other environmental or dietary genotoxicants are also likely to contribute to these mutations. Thus, specific chemical biomarkers of PhIP and other HAAs are required for molecular epidemiology studies investigating the role of cooked meat, HAAs, and cancer risk.

PhIP is the most abundant HAA carcinogen formed in well-done cooked meats. Currently, the PhIP hair biomarker is the only long-term HAA biomarker available and serves as a surrogate for estimating total HAA exposure. Many omnivores harbor PhIP in their hair, demonstrating that cooked meat consumption is prevalent, and exposure to PhIP (and other HAAs) is widespread. The PhIP hair biomarker proves that PhIP is bioavailable and undergoes systemic circulation. Biomonitoring PhIP in hair is expected to be a more reliable estimate of HAA exposure than the FFQ commonly used in epidemiology studies. However, the PhIP level in hair is a measure of exposure and biomarkers of the biologically effective dose, including HAA-DNA and HAA-protein adducts, should be implemented in epidemiology studies investigating the health risk of HAAs in cooked meats. Further development of PhIP-SA adduct biomarkers is warranted.

The large body of research shows that humans efficiently bioactivate HAAs to reactive intermediates. The major pathway of PhIP metabolism in humans occurs through CYP1A2 mediated N-oxidation to produce the reactive intermediate HONH-PhIP, which covalently binds to protein and DNA. The primary metabolites of PhIP in the urine of omnivores are Gluc conjugates of HONH-PhIP, demonstrating the critical role of UGT enzymes in PhIP metabolism. PhIP also induces cellular signaling pathways in human prostate cells at human dietary exposure levels, resulting in increased cell proliferation and cell migration, processes linked to the promotion and progression of neoplasia. Thus, PhIP may act as a tumor-initiator and promoter in the prostate and possibly other cancer target organs. Red meat contains a complex group of chemicals. Other chemicals formed during high-temperature cooking may contribute to cancer risk by acting as tumor initiators or promoters or by modulating carcinogen metabolism enzyme expression and activity, impacting the biological effects of HAAs. Future cohort studies implementing HAA biomarkers measured by specific mass spectrometry methods, combined with genetic polymorphisms in genes encoding carcinogen metabolism and DNA repair enzymes, can advance our knowledge on the health risks of dietary HAAs in human cancer.

## Data Availability

All generated data are included in this manuscript, or were obtained from peer-reviewed articles cited in the literature.

## Abbreviations

AMS: Accelerator mass spectrometry
AP: Adenomatous polyposis coli
AIAs: Aminoimidazoarenes
AAs: Aromatic amines
AR: Androgen receptor
CYP: Cytochrome P450
DHT: Dihydrotestosterone
FFQ: Food Frequency Questionnaire
GC-NICI-MS: Gas chromatography with negative ion chemical ionization-mass spectrometry
GST: Glutathione *S*-transferase
HAAs: Heterocyclic aromatic amines
Hb: Hemoglobin
IHC: Immunohistochemistry
LC/MS: Liquid chromatography/mass spectrometry
met-Hb: Methemoglobin
HbO_2_: Oxy-hemoglobin
PAH: Polycyclic aromatic hydrocarbons
ppb: Parts-per-billion
SA: Serum albumin
SULTs: Sulfotransferases
*t*-BuOOH: Tert-butyl hydroperoxide
UGTs: Uridine diphosphate-Glucuronosyltransferases
WGS: Whole-genome sequencing
ANL: Aniline
HONH-ANL: Phenylhdroxylamine
4-ABP: 4-aminobiphenyl
HONH-4-ABP: *N*-hydroxy-4-aminobiphenyl
IFP: 2-amino-1,6-dimethylfuro[3,2-*e*]imidazo[4,5-*b*]pyridine
AAF: *N*-acetyl-2-aminofluorene
AFB_1_: Aflatoxin B_1_
Trp-P^− 1^: 3-amino-1,4-dimethyl-5*H*-pyrido[4,3-*b*]indole
Trp-P-2: 3-amino-1-methyl-5*H*-pyrido[4,3-*b*]indole
Glu-P-2: 2-aminodiprido[1,2-*a*,3′,2′-*d*]imidazole
Glu-P-1: 2-amino-6-methyldiprido[1,2-*a*,3′,2′-*d*]imidazole
APNH: 9-(4′-aminophenyl)-9*H*-pyrido[3,4-*b*]indole
AMPNH: 9-(4′-amino-3-methylphenyl)-9*H*-pyrido[3,4-*b*]indole
IQx: 2-amino-3-methylimidazo[4,5-*f*]quinoxaline
I*g*Qx: 2-amino-3-methylimidazo[4,5-*g*]quinoxaline
7-MeI*g*Qx: 2-amino-3,7-dimethylimidazo[4,5-*g*]quinoxaline
7,9-DiMeI*g*Qx: 2-amino-3,7,9-trimethylimidazo[4,5-*g*]quinoxaline
MeIQx: 2-amino-3,8-dimethylimidazo[4,5-*f*]quinoxaline
HONH-MeIQx: *N*-hydroxy-2-amino-3,8-dimethylimidazo[4,5-*f*]quinoxaline
IQx-8-COOH: 2-amino-3-methylimidazo[4,5-*f*]quinoxaline-8-carboxylic acid
8-CH_2_OH-IQx: 2-amino-8-(hydroxymethyl)-3-methylimidazo[4,5-*f*]quinoxaline
MeIQx-*N*^2^-Gl: *N*^2^-(ß-1-glucosiduronyl)-2-amino-3,8-dimethylimidazo[4,5-*f*]quinoxaline
HON-MeIQx-*N*^2^-Gl: *N*^2^-(ß-1-glucosiduronyl)-2-(hydroxyamino)-3,8-dimethylimidazo[4,5-*f*]quinoxaline
MeIQx-*N*^2^-SO_3_H: *N*^2^-(3,8-dimethylimidazo[4,5-*f*]quinoxalin-2-yl-sulfamic acid
DMIP: 2-amino-1,7-dimethylimidazo[4,5-*b*]pyridine
TMIP: 2-amino-1,5,6-trimethylimidazo[4,5-*b*]pyridine
4,8-DiMeIQx: 2-amino-3,4,8-trimethylimidazo[4,5-*f*]quinoxaline
7,8-DiMeIQx: 2-amino-3,7,8-trimethylimidazo[4,5-*f*]quinoxaline
MeIQ: 2-amino-3,4-dimethylimidazo[4,5-*f*]quinoline
IQ: 2-amino-3-methylimidazo[4,5-*f*]quinoline
HONH-IQ: *N*-hydroxy-2-amino-3-methylimidazo[4,5-*f*]quinoline
IQ[4,5-b]: 2-amino-1-methylimidazo[4,5-*b*]quinoline
PhIP: 2-amino-1-methyl-6-phenylimidazo[4,5-*b*]pyridine
HONH-PhIP: *N*-hydroxy-2-amino-1-methyl-6-phenylimidazo[4,5-*b*]pyridine
HON-PhIP-*N*^2^-Gl: *N*^2^-(ß-1-glucosiduronyl-2-(hydroxyamino)-1-methyl-6-phenylimidazo[4,5-*b*]pyridine
HON-PhIP-*N*3-Gl: *N*3-(ß-1-glucosiduronyl-2-(hydroxyamino)-1-methyl-6-phenylimidazo[4,5-*b*]pyridine
PhIP-*N*^2^-Gl: *N*^2^-(ß-1-glucosiduronyl-2-amino-1-methyl-6-phenylimidazo[4,5-*b*]pyridine
PhIP-*N*3-Gl: *N*3-(ß-1-glucosiduronyl-2-amino-1-methyl-6-phenylimidazo[4,5-*b*]pyridine
MeAαC: 2-amino-3-methyl-9*H*-pyrido[2,3-*b*]indole
AαC: 2-amino-9*H*-pyrido[2,3-*b*]indole
HONH-AαC: 2-hydroxyamino-9*H*-pyrido[2,3-*b*]indole
dG-C8-APNH: *N*4′-(2′-deoxyguanosin-8-yl)-4-aminophenylnorharman
dG-C8-MeIQx: *N*-(2′-deoxyguanosin-8-yl)-MeIQx
dG-C8-4,8-DiMeIQx: *N*-(2′-deoxyguanosin-8-yl)-4,8-MeIQx
dG-*N*^2^-MeIQx: 5-(2′-deoxyguanosin-*N*^2^-yl)-MeIQx
dG-C8-IQ: *N*-(2′-deoxyguanosin-8-yl)-IQ
dA-*N*^6^-IQ: 5-(2′-deoxyadenosin-*N*^6^-yl)-IQ
dG-*N*^2^-IQ: 5-(2′-deoxyguanosin-*N*^2^-yl)-IQ
dG-N7-IQ: *N*-(2′-deoxyguanosin-7-yl)-IQ
dG-C8-MeIQ: *N*-(2′-deoxyguanosin-8-yl)-MeIQ
dG-C8-AαC: *N*-(deoxyguanosin-8-yl)-AαC
dG-C8-MeAαC: *N*-(2′-deoxyguanosin-8-yl)-MeAαC
dG-C8-PhIP: *N*-(2′-deoxyguanosin-8-yl)-PhIP
8-oxo-dG: 8-oxo-2′-deoxyguanosine
